# Differences in airway microbiome and metabolome of single lung transplant recipients

**DOI:** 10.1186/s12931-020-01367-3

**Published:** 2020-05-06

**Authors:** Nirmal S. Sharma, Grant Vestal, Keith Wille, Kapil N. Patel, Feng Cheng, Srinivas Tipparaju, Sultan Tousif, Mudassir M. Banday, Xin Xu, Landon Wilson, Viswam S. Nair, Casey Morrow, Don Hayes, Andreas Seyfang, Stephen Barnes, Jessy S. Deshane, Amit Gaggar

**Affiliations:** 1 Center for Advanced Lung Disease and Lung Transplantation, University of South Florida, Tampa, FL USA; 2 Division of Pulmonary, Critical Care & Sleep Medicine, University of South Florida/Tampa General Hospital, University of South Florida, Tampa, FL USA; 3 Division of Cardiothoracic Surgery, University of South Florida, Tampa, FL USA; 4 Brigham and Women’s Hospital, Harvard Medical School, Thorn-908 C, 20 Shattuck St, Boston, MA USA; 5 Division of Pulmonary, Allergy and Critical Care Medicine, University of Alabama at Birmingham, Birmingham, AL 35294 USA; 6 Department of Pharmaceutical Sciences, University of South Florida, Tampa, FL USA; 7 Program in Protease and Matrix Biology, University of Alabama at Birmingham, Birmingham, AL 35294 USA; 8 Metabolomics Core, Microbiome Core, University of Alabama at Birmingham, Birmingham, AL 35294 USA; 9 Division of Pulmonary, Critical Care & Sleep Medicine, University of Washington School of Medicine, Washington, USA; 10 Department of Pediatrics, The Ohio State University, Nationwide Children’s Hospital, Columbus, OH USA; 11 Department of Molecular Medicine, University of South Florida, Tampa, FL USA

## Abstract

**Background:**

Recent studies suggest that alterations in lung microbiome are associated with occurrence of chronic lung diseases and transplant rejection. To investigate the host-microbiome interactions, we characterized the airway microbiome and metabolome of the allograft (transplanted lung) and native lung of single lung transplant recipients.

**Methods:**

BAL was collected from the allograft and native lungs of SLTs and healthy controls. 16S rRNA microbiome analysis was performed on BAL bacterial pellets and supernatant used for metabolome, cytokines and acetylated proline-glycine-proline (Ac-PGP) measurement by liquid chromatography-high-resolution mass spectrometry.

**Results:**

In our cohort, the allograft airway microbiome was distinct with a significantly higher bacterial burden and relative abundance of genera *Acinetobacter* & *Pseudomonas*. Likewise, the expression of the pro-inflammatory cytokine VEGF and the neutrophil chemoattractant matrikine Ac-PGP in the allograft was significantly higher. Airway metabolome distinguished the native lung from the allografts and an increased concentration of sphingosine-like metabolites that negatively correlated with abundance of bacteria from phyla *Proteobacteria*.

**Conclusions:**

Allograft lungs have a distinct microbiome signature, a higher bacterial biomass and an increased Ac-PGP compared to the native lungs in SLTs compared to the native lungs in SLTs. Airway metabolome distinguishes the allografts from native lungs and is associated with distinct microbial communities, suggesting a functional relationship between the local microbiome and metabolome.

## Introduction

Recent studies have implicated the lung microbiome in the occurrence of chronic lung diseases such as idiopathic pulmonary fibrosis (IPF), chronic obstructive pulmonary disease, cystic fibrosis and chronic lung allograft dysfunction (CLAD) in lung transplant recipients [1–4]. There is emerging evidence that early alterations in lung microbiome and/or dysbiosis modulates inflammatory mediators leading to pathogenesis and/or progression of chronic lung diseases including CLAD [5–7]. Our group recently reported that a shift to a *Proteobacteria* dominant allograft microbiome was associated with CLAD in lung transplant recipients [8]. Likewise, microbial adaptations and changes in bacterial diversity have been implicated in progression of fibrosis in IPF subjects [4]. However, the mechanisms involved in the microbiome-host interactions leading to chronic lung inflammation are not well understood.

Delineation of the microbiome signatures and taxonomic profiles of bacterial communities in various disease states is an important first step but does not directly provide an insight into bacteriome-allograft-host interaction. Bacterial colonization and/or infection leading to pathology often results in physiological changes in the host, including alterations in metabolic profile [9, 10]. A better understanding of these metabolic shifts associated with a specific infection can improve our understanding of disease pathophysiology through the identification of by-products of host and microbial metabolism, while also providing vital information about the unique metabolites produced with these ever-changing interactions [11]. In particular, the delineation of bacterial metabolism and the host/allograft inflammatory response is critical to better define factors modulating the local microbiome. Metabolites or metabolomic profiles identified in a defined cohort can also serve as potential biomarkers for disease characterization and/or novel therapeutic targets [12]. In the context of a potential clinical application, unique metabolome signatures in urine have been found to distinguish *Streptococcus pneumoniae* from *Staphylococcus aureus* lung infection [13]. Similarly, lung metabolome analysis from HIV subjects have shown that pyochelin, a siderophore produced by *Pseudomonas aeruginosa*, is elevated in HIV-infected individuals compared to HIV-uninfected individuals [14]. Likewise, specific metabolome pathways have been identified in lung transplant recipients with CLAD [15], though further investigations are needed to delineate its relevance to CLAD pathobiology. Identification and correlation of novel metabolome profiles associated with specific pathological microbiome signatures may help guide personalized treatment of host disease states [16].

Dysregulation of muco-ciliary clearance and subsequent increases in bacterial burden are well described in advanced lung disease [17, 18]. Moreover, colonization of pathobionts in diseased native lungs of single lung transplant recipients (SLTs) may alter and/or contribute to the microbiome of the allografts [19, 20]. However, to date, the interaction of the metabolome and microbiome in the allografts and native lungs has not been evaluated in patients after lung transplantation. In this study, we utilized the airway microbiome and metabolome signatures of the native lungs and transplanted lungs (allografts) of SLTs as a model system to answer fundamental questions regarding the inherent lung metabolome and its influence on the lung microbiome. We hypothesized that the airway metabolome would correlate with the abundance of distinct microbiome signatures in the native and allografts lungs of SLTs.

## Material and methods

Subjects for the study were recruited from the adult lung transplant program at the University of Alabama at Birmingham between September 2014 to July 2016. Six consecutive adult (> 18 years) single lung transplant recipients undergoing bronchoscopy due to a decline in pulmonary function were recruited for this study. Written consent was obtained for sample collection under an institutional review board–approved protocol (IRB No. X120606006, University of Alabama at Birmingham). Subject details are elucidated in Table 1. Bronchoalveolar lavage (BAL) samples from non-transplant volunteers attending the UAB lung health center clinic and University of South Florida were collected. Demographics of the non-transplant volunteers are detailed in supplementary text.

**Table 1:** Baseline Characteristics of Single lung transplant subjects

S. No	Age	Gender	Side of Transplant	Duration of Transplant (months)	Pre-transplant diagnosis	IS	Prophylactic Antibiotic	BAL Culture	BAL cell count differential	CLAD at sample collection (Y/N)	CLAD at follow-up (2 years) (Y/N)
**1**	**64**	**M**	**Left**	**20**	**IPF**	**Tac, Pred, Aza**	**Val, Itra, Dapsone,**	**Negative**	**M-99%, N-1%**	**N**	**N**
**2**	**57**	**M**	**Right**	**13**	**CTD-ILD**	**Tac, Pred, MMF**	**TMP/SMX, Val, Vori**	**Negative**	**M-95%, L-3%, N- 2%**	**Y**	**Y**
**3**	**64**	**M**	**Left**	**30**	**IPF**	**Tac, Pred, MMF**	**Azithro, TMP/SMX, Val**	**Negative**	**M-10%, L-40%, N- 50%**	**N**	**Y**
**4**	**69**	**M**	**Left**	**31**	**IPF**	**Tac, Pred, Aza**	**Dapsone, Azithro,**	**Negative**	**M-93%, L-5%, N-2%**	**N**	**Y**
**5**	**59**	**F**	**Right**	**18**	**IPF**	**Tac, Aza Pred**	**TMP/SMX**	**Negative**	**M-38%, L- 54%, N 8%**	**N**	**Y**
**6**	**62**	**M**	**Left**	**79**	**IPF**	**Tac, Pred**	**Dapsone**	**1000 CFU normal flora**	**M-58%, L-3%, N-39%**	**Y**	**Y**

*Abbreviations*: *IS* Immunosuppression, *Azithro* Azithromycin; *CLAD* Chronic Lung Allograft Dysfunction, *IPF* Idiopathic pulmonary fibrosis, *CTD-ILD* Connective tissue disease related interstitial lung disease, *Aza* Azathioprine, *Itra* itraconazole, *MMF* Mycophenolate mofetil, *Pred* Prednisone, *Tac* Tacrolimus, *TMP/SMX* Trimethoprim–sulfamethoxazole, *Val* Valganciclovir, *Vori* Voriconazole, *M* Macrophages, *L* Lymphocytes, *N* Neutrophils

### Sample collection

Bronchoalveolar lavage (BAL) fluid from the allograft (A) (i.e. the transplanted lung) and the native lung (N) of each SLT subject were collected using two separate bronchoscopes. Bronchoscopic control samples (C) were collected from each bronchoscope used prior to the procedure (25 ml of sterile saline flushed via the bronchoscope and collected). For standardization, all bronchoscopies were conducted via the oral route and separate scopes used for sampling the A and N sides of each subject. A total of 120 ml of saline (4 aliquots of 30 cc) were instilled for the BAL on each side (right middle lobe and lingula) and remnant BAL fluid was collected from the last aliquot fraction from the right middle lobe and lingula of allograft and native lung. Similarly, non-transplant normal lung volunteers BAL (H) were collected and processed.

### Processing of samples

Two aliquots (5 ml each) of **N, A** and **H** BAL fluid samples were centrifuged at 1000 rpm for 5 min to separate the eukaryotic cellular fraction. The supernatants were centrifuged again at 15,000 rpm for 10 min to pellet the bacterial component. Similarly, 2 aliquots (5 ml each) of control (C) samples from each bronchoscope used were collected and centrifuged at 15,000 rpm for 10 min to pellet the bacterial component. All bacterial pellets were then stored at − 80 °C. Supernatants from each samples after collection of bacterial pellet were also stored at − 80 °C. DNA was extracted from the bacterial pellets. The two processed aliquots from each BAL sample served as technical replicates. Microbiome sequencing as below was performed from each BAL sample in duplicates. DNA from pellets was extracted using Zymo DNA extraction (CA) kit. Polymerase chain reaction (PCR) amplification of the V4 region of the 16S rRNA gene was performed and products used for microbial DNA sequencing using illumina miseq platform. Data was analyzed used QIIME pipeline and CLC genomics workbench platforms [8]. 16S quantitative PCR to estimate bacterial count was performed using published methodologies [21–23]. Supernatant from the above samples were used to measure Ac-PGP levels, multiplex cytokine assays and untargeted metabolome analysis using liquid chromatography-high resolution mass spectrometry using established techniques. Details of our experimental protocols are provided in the supplementary materials.

### Statistical analysis

To compare β-diversities between individual patient allograft and native lung samples, weighted UniFrac distances were calculated between all pairs of samples and then each sample type was plotted separately in 3-dimensional (3D) space by principal coordinate analysis. The plots were then transformed by Procrustes analysis to achieve maximum alignment. Within the 3D plots, blue color represents 1 sample and the red color represents the other sample, and the 2 points from individual subjects are connected by a bar. If both plots are similar, then the relative distance will be small. The overall similarity is summarized by the M^2^ value, and statistical goodness-of-fit is measured by a Monte Carlo label permutation approach. To identify individual OTUs at the phylum and/or genus levels that were distinctive between the 2 airway compartments in native and allograft, wilcoxon test (non-parametric). Differences between the native, allograft and normal was calculated using non-parametric ANOVA (Kruskal Wallis) with subgroup analysis conducted by Dunn multiple comparison test. Significant differences in community membership identified via constrained ordination were confirmed by using PERMANOVA (permutational multivariate analysis of variance) and plotted in R package vegan via RDA function.

Features in the LC-MS metabolomics data were aligned and peak areas determined using XCMSonline (https://xcmsonline.scripps.edu/) [24]. Statistical analysis of peak areas was carried using the univariate (Volcano plot) and multivariate (sparse Partial Least Squares-Discriminant Analysis, sPLS-DA) programs of MetaboAnalyst (http://www.metaboanalyst.ca) [25]. Metlin (https://metlin.scripps.edu/) [26] was used to identify the individual *m/z* features. Mummichog (http://www.mummichog.org, version 1.0.9) [27] was used for pathway analysis. Fischer’s Exact test was used for comparison and generation of enrichment *P* values. *P*-values for all pathways were then modeled as a Gamma distribution and then adjusted for the permutations. The correlations and interaction plot between proportions of a metabolome and a bacterial phylum were generated using the online software XMWAS (https://kuppal.shinyapps.io/xmwas/) [28]. GRAPHPAD PRISM version 7.0 (GraphPad, Inc., San Diego, CA software) was used for statistical analysis and generation of figures, with statically significance achieved at *p* value = 0.05.

## Results

### Differences between the allograft and native lung microbiome

**Fig. 1:**
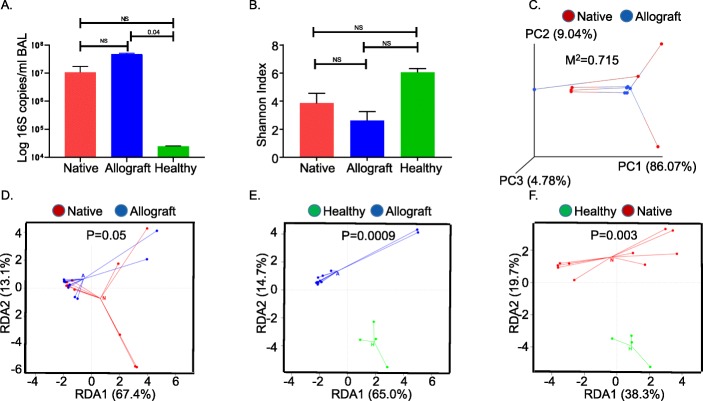
**a** Bacterial 16S gene copies/ml in allograft (A), native (N) of single lung transplant recipients and normal (H) lung controls. The Y axis indicates 16S rRNA gene copy number by quantitative PCR. (N vs A vs H, P = 0.02 (Kruskal Wallis), post-hoc Dunn’s test, N vs A = 0.1, N vs H = NS, A vs H = 0.04,) (**b**) Shannon diversity index in allograft (A), native (N) of single lung transplant recipients and normal (H) lung controls. N vs A vs H, P = 0.06 (Kruskal Wallis), post-hoc Dunn’s test, N vs A = NS, N vs H = NS, A vs H=NS (**c**) Relationship between native and transplant airway bacterial communities within individual subjects. Weighted UniFrac distances were calculated between all pairs of samples within native or allograft, and then each sample type was plotted separately in 3D space by principal coordinate analysis. The two plots (allograft and transplant) were then transformed by Procrustes analysis to achieve maximum alignment. Each point corresponds to a bacterial community, with native communities shown in *red*, allograft communities shown in *blue*, and the two communities from each subject connected by a *bar*. The *black* end of each bar connects to the native sample data; the *grey* end connects to the allograft lung sample data from the same individual. If native and allograft plots are similar, then the relative distance between connected points (residuals) will be small. The overall similarity is summarized by the M^2^ value, and statistical goodness of fit is measured by a Monte Carlo label permutation approach (10,000 iterations). The M^2^ value ranges from 0 to 1, with 0 suggesting complete overlap i.e. similarity and 1 suggesting maximum variation. M^2^ = 0.715 suggests a greater degree of variation between the native and allograft microbiome from individual patients. **d** Principal coordinate analyses (PCoA): ordination constrained by specimen group (RDA) showing spatial relationship of the variance between allograft (A) and native (N) of single lung transplant recipient microbiomes. Native are shown in *red and* allograft in *blue.* P value calculated via PERMANOVA analysis N vs A, *P* = 0.05. **e** Principal coordinate analyses (PCoA): ordination constrained by specimen group (RDA) showing spatial relationship of the variance between allograft (A) and normal (H) lung microbiomes. Normal lung controls are shown *in green* and allograft in *blue. P* value calculated via PERMANOVA analysis A vs H, *P* = 0.0009 (**f**) Principal coordinate analyses (PCoA): ordination constrained by specimen group (RDA) showing spatial relationship of the variance between native (N) lung of single lung transplant recipients and normal (H) lung control microbiomes. Normal lung controls are shown *in green* and native in *red.* P value calculated via PERMANOVA analysis N vs H, *P* = 0.003

We first determined the bacterial count in Allograft (A), Native (N), normal (H) groups using 16 S quantitative PCR. Overall, bacterial 16S DNA levels reported as log 16S copies/ml of BAL were significantly different among the groups (*P* = 0.02, A 4.15 × 10^7^ vs N 2.0 × 10^7^, *P* = 0.1, A 4.15 × 10^7^ vs H1.8 × 10^5^, *P* = 0.04, N vs H, P=NS) and higher in the allograft compared to normal controls (Fig. 1a). Next, to determine whether A, N and H differed in diversity of bacterial communities between samples, we used the Shannon diversity index [29], a measure of α-diversity within a sample that represents both species richness and evenness (Fig. 1b). A Shannon diversity index of ≥3.5 indicates a highly diverse bacterial community [30]. Although shannon diversity index of the allograft was lower (2.4) compared to was higher in H (5.38) and N (3.8) samples, these were not statistically different (*P* = 0.06).

We evaluated the differences in the microbiome compositions between A and N of each individual lung transplant recipient. Using Procrustes analysis, we mapped individual samples on a principal component analyses (PCoA) plot using weighted UniFrac distances. The PCoA showed wide separation between A and N microbiomes of each individual subject (Fig. 1c). Monte Carlo label permutation was used to calculate a M^2^ value. The M^2^ value varies between 0 to 1 and a higher number suggests greater variation between the samples groups. The M^2^ value was 0.715 suggesting a wide variation between the A and N samples derived from individual patients. In addition, the pooled analysis using redundancy analysis (RDA) showed a cluster pattern for A versus the N microbiomes. Significant differences in community membership between the constrained ordination plot were confirmed using PERMANOVA, *P* = 0.05 (Fig. 1d). When comparing the A and N microbiome to normal lung microbiome (H), again a distinct clustering pattern was noted for each group (PERMANOVA H vs A, *P* = 0.0009, H Vs N, *P* = 0.003, Fig. 1e and f). Significant microbiome signature differences between the A and N groups are detailed in Table ST1 in the supplemental materials.

To confirm the validity of our findings and rule out contamination from the bronchoscope, we assessed the microbial signatures present in the bronchoscope washes (C) collected prior to the collection of BAL fluid samples. PCoA analysis showed significant weighted UniFrac distances between the C microbiome of the bronchoscopes when compared to their respective N and A microbiome of individual subjects (Figure S1A and B and Tables ST2 and ST3 in Supplementary Material).

### Allograft lung has a higher abundance of genera *Acinetobacter, Pseudomonas* and increased ac-PGP

**Fig. 2:**
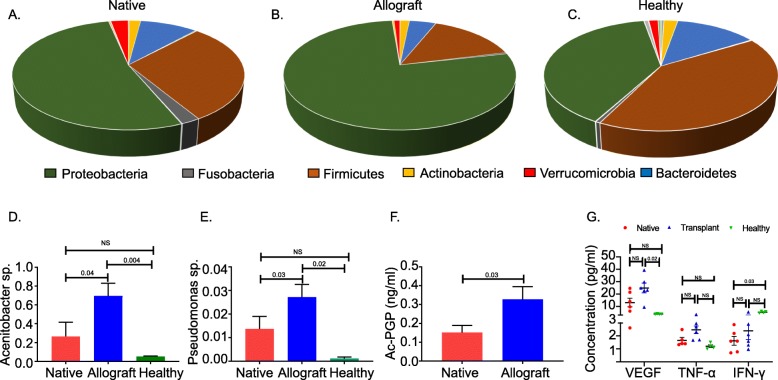
Composition of major bacterial phyla in the bronchoalveolar lavage samples from (**a**) native lungs of SLTs (**b**) allograft lungs of SLTs and (**c**) normal lung subjects (**d**) Relative abundance of genus *Acinetobacter* in the bronchoalveolar lavage samples from allograft (A), native (N) of single lung transplant recipients and normal (H) lung controls. N vs A vs H, P = 0.003 (Kruskal Wallis), post-hoc Dunn’s test, N vs A = 0.04, N vs H = NS, A vs H = 0.004 (**e**) Relative abundance of genus *Pseudomonas* in the bronchoalveolar lavage samples from allograft (A), native (N) of single lung transplant recipients and normal (H) lung controls. N vs A vs H, P = 0.01 (Kruskal Wallis), post-hoc Dunn’s test, N vs A = 0.03, N vs H = NS, A vs H = 0.02. **f** Concentration of Ac-PGP in the bronchoalveolar lavage samples from native lungs and allografts of SLTs, *P* = 0.03 (Wilcoxon test) (**g**) Pro-inflammatory cytokines VEGF, TNF-α, and IFN-γ in the bronchoalveolar lavage samples from native lungs, allografts of SLTs and normal controls. VEGF A vs N vs H, *P* = 0.002, Post-hoc Dunn’s test, N vs A = NS, N vs H = NS, A vs H = 0.002, TNF-α A vs N vs H, *P* = 0.03, Non-significant subgroup comparisons, IFN-γ A vs N vs H, *P* = 0.01, Post-hoc Dunn’s test, N vs A = NS, N vs H = 0.03, A vs H = NS

Emergence of microbiome dominated by a single and/or a group of bacteria, gives rise to microbial dysbiosis in the gut and lung; this is associated with immune dysregulation and has been linked to several chronic inflammatory disease states [31, 32]. The healthy lung microbiome is largely composed of the phyla *Firmicutes, Bacteroidetes and Proteobacteria* [33]. To understand the local microbiome of the native lung and allograft, we studied the overall community composition structure in samples from the allograft of SLTs and compared them to the corresponding native lung microbiome. In addition, we analyzed their differences with the normal lung microbiome. Overall, the relative abundance of Phyla *Proteobacteria* (A 77% vs N 52% vs H 39%, *p* = 0.02) and *Firmicutes* (A 14% vs N 29% vs H 41%, *p* = 0.009) were significantly different between the allograft, native and normal lung controls (Fig. 2a, b, c). Previously, an increased *Firmicutes/Bacteroidetes* (F/B) ratio has been suggested as a marker for dysbiosis in the gut [34]. We calculated the F/B ratio in our cohorts. Additionally, we calculated the *Proteobacteria/Firmicutes* (P/F) ratio given that several members of the phyla *Proteobacteria* have been implicated in the pathogenesis of respiratory diseases*.* In our sample both F/B (A (3.25) vs N (2.8) vs H (2.9)) ratio and P/F (A (5.5) vs N (1.7) vs H (1.4)) were higher in the allografts compared to both the native and healthy lung controls. At the genera level, *Acinetobacter* and *Pseudomonas* were significantly higher in the allografts compared to both the native and normal lung controls (Fig. 2d and e). The hospital-based BAL cultures were negative for *Pseudomonas,* Acinetobacter or any other significant pathogenic bacteria (Table 1).

Next, we measured the levels of Ac-PGP, a collagen breakdown matrikine peptide that is known to upregulate neutrophil chemotaxis and increase lung vascular permeability [35, 36]. We found that Ac-PGP was elevated (*p* = 0.03) in the allograft as compared to native lungs (Fig. 2f). Additionally, we measured other neutrophilic chemoattractants, IL-8 and LTB4 in the native and allograft samples and these were not found to be statistically different between the two groups (supplementary Figure 2A and B). Thereafter, we measured the levels of pro-inflammatory cytokines (VEGF, TNF -α, IFN-γ) in the BAL. Overall, the levels of all three cytokines were statistically different between A, N and H. Subgroup analysis showed that VEGF was significantly increased in the A compared to H. In contrast, the levels of IFN-γ were reduced in N compared to H (Fig. 2g).

### Airway metabolome distinguishes native lungs and allografts

**Fig. 3:**
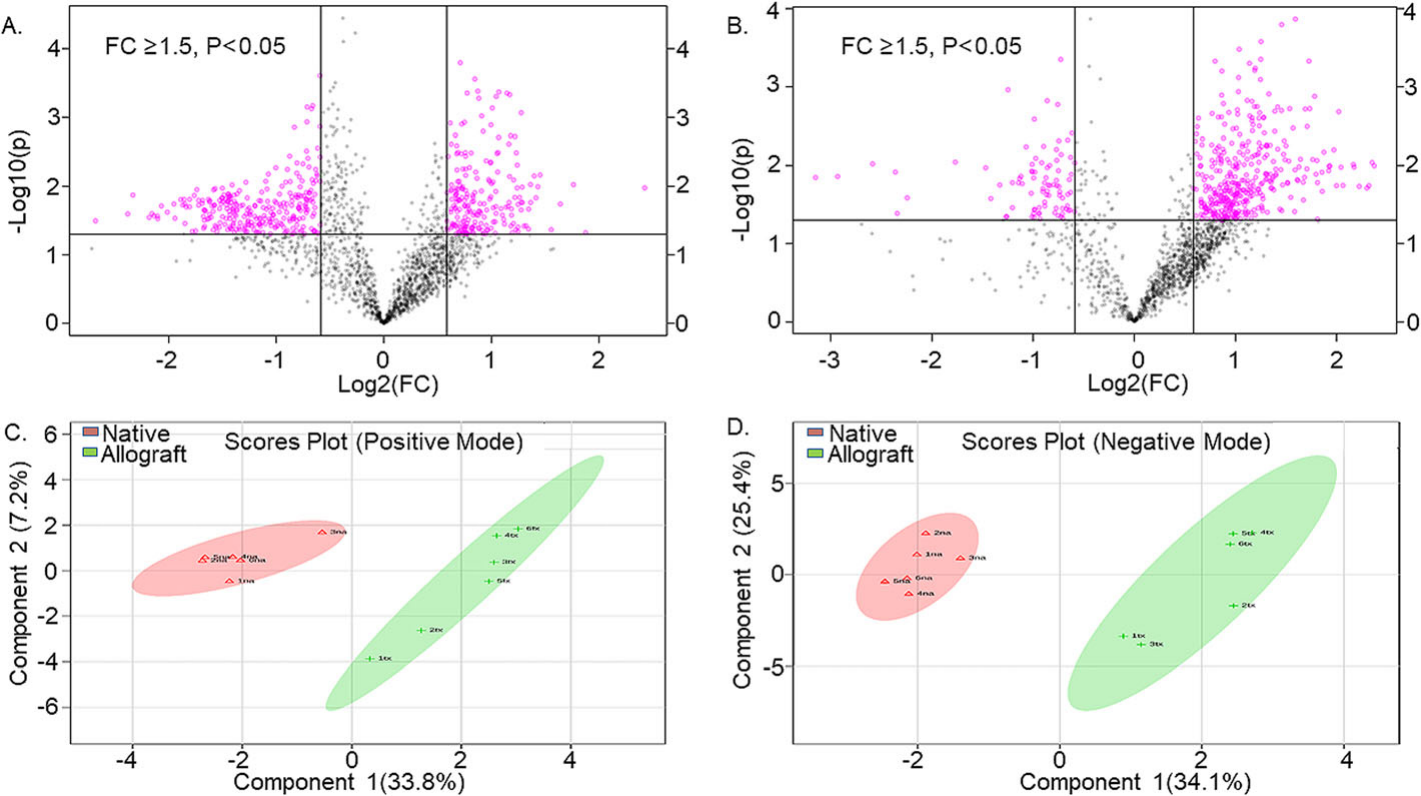
**a** Volcano plot in positive ion mode showing the statistical significance (y axis) and fold change (x axis) for difference between the metabolome of native and allograft lung bronchoalveolar lavage samples. P < 0.05, Fold change ≥1.5 (**b**) Volcano plot in negative ion mode showing the statistical significance (y axis) and fold change (x axis) for difference between the metabolome of native and allograft lung bronchoalveolar lavage samples. *P* < 0.05, Fold change ≥1.5 (**c**) Score plots of principle component analysis of metabolome from native and allograft bronchoalveolar samples in the positive ion modes. These plots display a clear separation between native and transplant metabolome. The color circle (green and red) around each sample group represents the 95% confidence intervals. **d** Score plots of principle component analysis of metabolome from native and allograft bronchoalveolar samples in the negative ion modes. These plots display a clear separation between native and allograft metabolome. The color circle (green and red) around each sample group represents the 95% confidence intervals

Airway metabolome can provide an insight in to the inflammatory state of the lung [37]. Using an untargeted metabolomics approach and analysis previously described [38, 39], we identified a total of 4886 mass/charge (*m/z)* features in LC–MS data collected in both the negative ion and positive ion modes. Of these, 676 *m/z* features in the negative ion mode and 586 *m/z* features that were significantly different (*p* < 0.05) between the allografts and native lungs. After reorganizing the downloaded LC–MS data from XCMSonline to remove early (non-bound) metabolite ions and ions collected in the solvent wash period, a volcano plot (Fig. 3a, b) revealed several negatively charged ions and positively charged ions with fold changes > 1.5 and *p*-values < 0.05. PCoA and PLS-DA analyses were performed to assess chemometric separation among the allograft and native lung BAL fluid samples. A sparse PLS-DA plot showed complete separation of the allograft and native lung BAL samples in both the negative and positive ion mode (Fig. 3c and d).

The molecular identity of the top variable importance in projection (VIP) score metabolites (Figure S3A, B) observed by nanoLC–MS that were significantly changed in the negative and positive ion mode were determined based on their measured accurate mass using the Metlin database (https://metlin.scripps.edu/) or by fragmentation pattern of the ion. The *m/z* features 332.278, 316.283, 296.257, 314.268, 298.273 were identified as sphingosine like metabolites and found to be significantly increased in the native lungs compared to the allograft (Figure S3A and Table 2).

**Table 2:** Table of differentiating metabolites by VIP score between native and allograft BAL samples (matched in Metlin at 10PPM or by fragmentation pattern of the ion)

Input m/z	Adduct	Actual Mass	Database Match	Formula
184.133	(M + H)^+^	183.126	Acetyltropine	C10H17NO2
332.278	(M + H)^+^	331.272	Sphingosine like molecule	C18H37NO4
202.143	(M + H)^+^	201.137	Capryloyglycine	C10H19NO3
316.283	(M + H)^+^	315.277	Dehydroxysphingosine	C18H37NO3
296.257	(M + H)^+^	295.251	(4E,8E,10E-d18:3) sphingosine	C18H33NO2
314.268	(M + H)^+^	313.262	Sphingosine like molecule	C18H35NO3
186.115	(M-H)^−^	187.121	(E)-2-Butenyl-4-methyl-threonine	C9H17NO3
201.1	(M + H)^+^	200.12	N-(5-Methyl-3-oxohexyl) alanine	C10H19N03
339.2682	(M + H)^+^	338.2610	Androstane	C24H34O

Metabolite annotation and pathway enrichment analysis were performed using mummichog analysis to understand the role of the identified ions in different metabolic pathways. Mummichog identified 21 pathways associated with the negative ion metabolites that were found in the native and allograft BAL fluid samples (Table 3). Metabolites identified were known to be involved in fatty acid activation/metabolism, beta-oxidation, Krebs cycle and amino acid and nucleotide metabolism.

**Table 3:** Metabolite annotation and pathway enrichment analysis using Mummichog for negative ion m/z features present in the native and allograft BAL fluid. * *P* < 0.05, Native vs Allograft samples with less enrichment of these pathways. Fischer’s Exact test was used for comparison and generation of enrichment P values. P-values for all pathways were then modeled as a Gamma distribution and then adjusted for the permutations

Pathways	Pathway Size	Adjusted ***P*** Value
**Purine Metabolism**	**22**	**0.001**
**Methionine and Cysteine Metabolism**	**13**	**0.002**
**Denovo-Fatty acid biosynthesis**	**10**	**0.002**
**Fatty acid activation**	**7**	**0.003**
**Vitamin B5-CoA biosynthesis**	**7**	**0.003**
**Aspartate and Aspargine Metabolism**	**21**	**0.003**
**Alanine and Aspartate Metabolism**	**5**	**0.005**
**Arginine and Proline Metabolism**	**9**	**0.006**
**Drug Metabolism CYP 450**	**33**	**0.008**
**Saturated fatty acid beta-oxidation**	**2**	**0.008**
**Phosphatidylinositol Phosphate Metabolism**	**6**	**0.009**
**TCA Cycle**	**3**	**0.01**
**Alkaloid Biosynthesis II**	**3**	**0.01**
**Urea cycle**	**20**	**0.01**
**Pyrimidine Metabolism**	**13**	**0.03**
**Dynorphin Metabolism**	**4**	**0.03**
**Omega 3 Fatty acid Metabolism**	**4**	**0.03**
**COA Metabolism**	**4**	**0.03**
**Fatty acid Metabolism**	**4**	**0.03**
**Vitamin E Metabolism**	**27**	**0.03**
**Linoleate Metabolism**	**9**	**0.03**

### Sphingosine metabolites negatively correlate with abundance of *Proteobacteria*

**Fig. 4:**
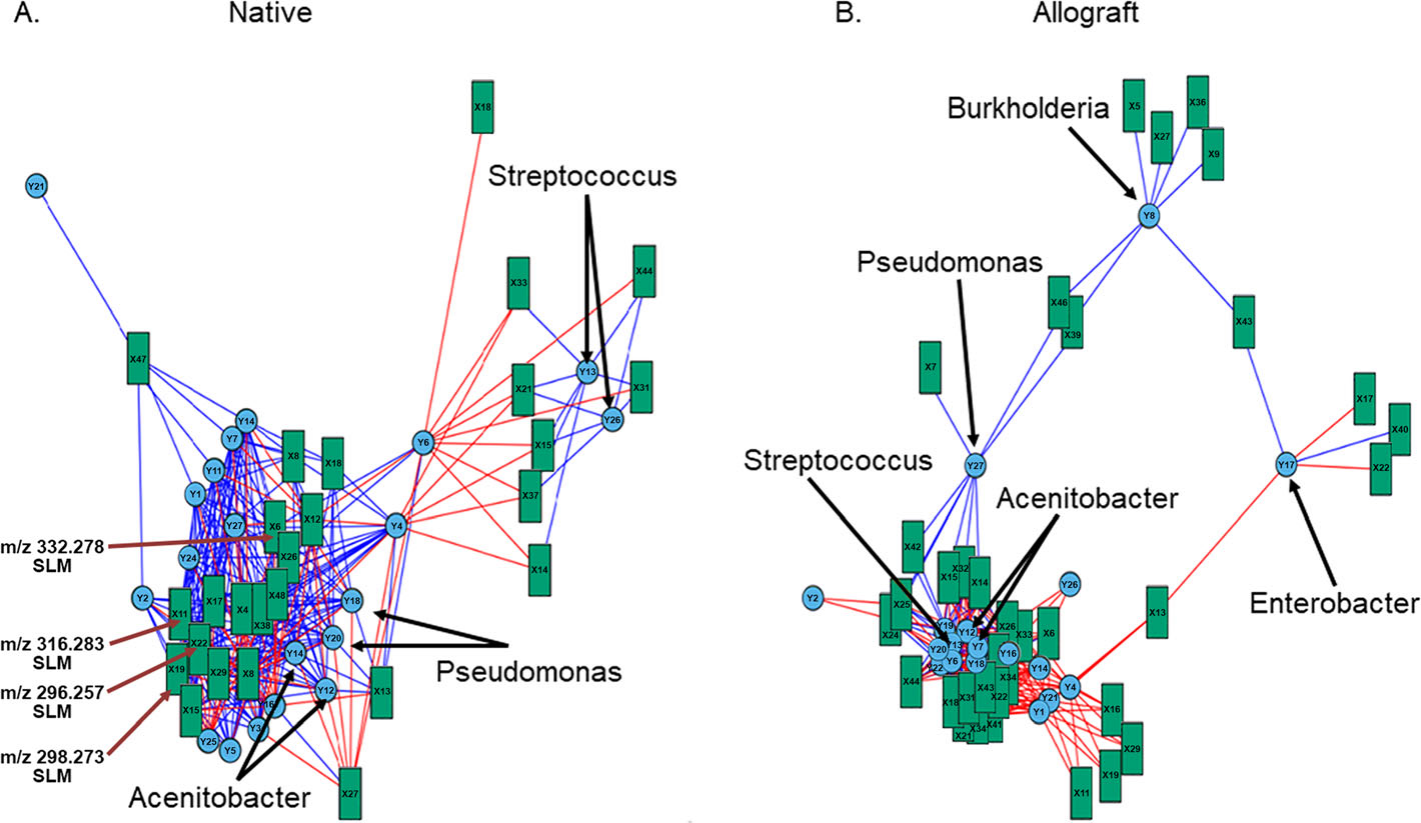
**a** Multidata network plot with the communities identified using the multilevel community detection algorithm. Correlation networks of bacterial genus and metabolome m/z features of the native lung bronchoalveolar lavage. Metabolome features are shown in green squares and microbiome measurements of genera level OTU in blue circles. Links indicate pairwise Pearson’s correlations, |*r*| > 0.40. Red links indicate a positive correlation, whereas blue links indicate a negative correlation. Thickness of the link indicates the strength of the pairwise correlations. Sphingosine like molecules (SLM) with significant negative correlation with microbiome features are highlighted. Each green square has an encoded number starting with X that represents the metabolite found to have significant correlation with a corresponding microbiome feature (encoded in Y) in blue circles. Details of the X and Y number notations in native lung can be found in table ST6. **b** Multidata network plot with the communities identified using the multilevel community detection algorithm. Correlation networks of bacterial genus and metabolome m/z features of the allograft bronchoalveolar lavage. Metabolome features are shown in green squares and microbiome measurements of genera level OTU in blue circles. Links indicate pairwise Pearson’s correlations, |*r*| > 0.40. Red links indicate a positive correlation, whereas blue links indicate a negative correlation. Thickness of the link indicates the strength of the pairwise correlations. Each green square has an encoded number starting with X that represents the metabolite found to have significant correlation with a corresponding microbiome feature (encoded in Y) in blue circles. Details of the X and Y number notations in the allograft can be found in table ST7

To understand the correlation between the microbiome and metabolome, we performed integration analysis using sparse PLS-DA regression of all *m/z* features (4885) and the 16 Phyla found in the native lung and allograft samples. We found that overall 864 *m/z* features were associated with the various bacterial phyla [16] at a correlation of > 0.4. The phyla *Proteobacteria, Firmicutes and Bacteroidetes* had positive and negative correlations with several *m/z* features both in the native and allograft lungs (Table ST4, ST5). Next, we performed integration analysis of the top 25 *m/z* features by VIP score each in the positive and negative mode and the significantly different microbial genera [28] between the two groups. The VIP score is a measure of a variable’s importance in the PLS-DA model. The VIP score of a variable is calculated as a weighted sum of the squared correlations between the PLS-DA components and the original variable. It summarizes the contribution a variable makes to the model. Of the top 50 metabolites by VIP score in the positive and negative mode, 35 *m/z* features correlated with 18 bacterial genera in the allografts and 26 *m/z* features correlated with 20 bacterial genera in the native lung. In the native lungs, the genera associated with most features at a correlation ≥0.4 were *Acinetobacter* (22 features) and Pseudomonas (17 features) (Fig. 4a). Whereas in the allografts, the genera associated with the greatest number of features at a correlation ≥0.4 were *Acinetobacter* (18 features), Pseudomonas (17 features), *Bacteroidetes* (18 features) and Streptococcus (17 features). The genera *Burkholderia, Enterobacter* and some of their associated *m/z* features were in a separate network compared to the others as illustrated in the network plot 4B. In the native lungs, several sphingosine-like metabolites (e.g., *m/z* 296.257, 298.273, 314.268) had a negative correlation with bacterial genera (*Pseudomonas, Acinetobacter*) from the phyla *Proteobacteria* (Table ST6, Fig. 4a). Similarly, amino acid metabolites m/z features (201.1, 186.115) had positive correlation with genus *Acinetobacter* and *Pseudomonas* in the allograft lung.

## Discussion

Emerging evidence suggests a role for the lung microbiome in the pathogenesis of chronic inflammatory lung diseases including CLAD [5, 8, 40]. In this study, we utilized the airway microbiome and metabolome signatures of the native lungs and transplanted lungs (allografts) of SLTs as a model system to answer fundamental questions regarding the inherent lung metabolome and its influence on the lung microbiome. We characterized the associations between the airway microbiome and metabolome of the allograft and the native lungs of SLTs. In our cohort, we found that the airway microbiome of the native and allograft lungs were distinct with a significantly higher abundance of genus *Pseudomonas* and *Acinetobacter* (phyla *Proteobacteria)* and elevated levels of VEGF and ac-PGP in the allograft. Furthermore, the native lung metabolome differed from the allograft with a higher abundance of sphingosine and sphingosine-like metabolites and its presence negatively correlated with the abundance of bacteria *Pseudomonas* and *Acinetobacter*. These hypothesis generating observations lay the foundation for future studies to evaluate the cause-effect relationship between the airway metabolome and microbiome.

To our knowledge, this is the first report characterizing the microbial heterogeneity of the native and transplanted lung in the same individual. The results from our cohort suggests that the allograft’s airway microbiome is distinct with a significantly increased relative abundance of genera *Acinetobacter* & *Pseudomonas (*phyla *Proteobacteria*) (Figs. 1a, 2d and e). In comparison, the microbiome in native lungs of SLTs and healthy individuals have a greater proportion of the phyla *Firmicutes and Bacteroidetes*. Alterations in the *Firmicutes/Bacteroidetes* (F/B) ratio have previously been proposed as a marker for intestinal dysbiosis in various disease states [34]. In the lung, where a higher proportion of *Proteobacteria* is present than the gut [33], the *Proteobacteria/Firmicutes* (P/F) ratio may be a better marker for dysbiosis, given the predominance of *Firmicutes* in the healthy lung [33]. In our cohort, the allografts had an elevated F/B and P/F ratios compared to the native lungs and normal lung controls suggesting greater dysbiosis. Donor lungs at the time of implantation harbor a different microbiome than the recipient, indicative of greater microbial variation between the allograft and native lung at the time of transplantation. Following transplantation, common thinking is that over time the microbiomes of the allograft and native lung would become similar. However, in our cohort, despite the subjects being more than a year post-lung transplantation, the microbiome remained varied between the allograft and native lung. Likewise, we noted an increased bacterial biomass burden and dysbiosis in the allografts compared to the native and healthy lung controls. These results are concordant with other studies linking elevated bacterial biomass to microbial dysbiosis [41]. Increased bacterial biomass and colonization with pathogenic bacteria such as *Acinetobacter* and *Pseudomonas* has been associated with allograft dysfunction in lung transplant recipients [42].

Cytokine VEGF and tripeptide Ac-PGP were found to be significantly elevated in allograft BAL samples. We and others have shown that Ac-PGP, a matrikine tripeptide, mediates inflammation in acute and chronic lung diseases including CLAD and bacterial infections [43, 44]. Interestingly, elevated VEGF levels has been linked to lung infections with *Pseudomonas* [45] as well as allograft rejection including post lung transplant primary graft dysfunction and bronchiolitis obliterans syndrome, a form of chronic lung allograft dysfunction [46–48]. Likewise, dysbiosis in the lung and gut has been implicated in heightening inflammatory states in several chronic diseases [31, 32]. The increase in the ratio of the pro-inflammatory bacteria such as *Pseudomona*s and low stimulatory bacteria such as *Prevotella* and *Streptococcus* are known to upregulate the inflammatory gene expression profile [5]. Although we see an association between elevated *Proteobacteria* the inflammatory markers Ac-PGP and VEGF, additional investigations need to be conducted to establish a causal link between a *Proteobacteria*-dominant microbiome and pro-inflammation.

To further understand the functional impact of the microbiome on the host, we conducted metabolome analyses. The airway metabolome differentiated the allograft and the native lung of the participants of our study (Fig. 3b and c). Several of these metabolites had significant negative and positive correlations with various bacterial genera (Fig. 4a, b). In the positive ion mode, the top m/z features that differentiated the allograft from the native lung (high VIP scores) were dominated by sphingosine-like molecules that were found to be increased in the native lungs (Table 2 and Figure S3A &B). These sphingosine metabolites had a negative correlation with bacterial genera (*Pseudomonas, Acinetobacter*) from the phyla *Proteobacteria* in the native lungs. Sphingolipids are bioactive lipids known to be part of the plasma membrane lipid bilayer in eukaryotic cells [49] and are cleaved by sphingosine kinases, with several of these molecules having key roles in the regulation of oxidative stress and immune function [50]. Sphingosine improves the host response to *Pseudomonas* infections and augments neutrophil killing of reactive oxygen species resistant *Pseudomonas* [51–53]. An increased presence of sphingosine-like molecules in the metabolome of native lungs may account for the lower *Proteobacteria* signature found in them as compared to the allografts. Likewise, some metabolite features had positive correlation with the microbial genera (*Pseudomonas* and *Acinetobacter*) in the allograft lungs. Metabolite m/z 201.1, a alanine metabolite and m/z 186.115, a threonine metabolite are involved in maintenance of bacterial cell wall structure and cellular stiffness, promote bacterial proliferation [54, 55] and have regulatory role in T cell activation [56]. Mummichog analysis of the metabolome suggested the presence of metabolites that were increased in the allograft compared to the native lung (Table 3). Pathways in the amino acid metabolism, fatty acid activation and fatty acid beta-oxidation were increased in the allograft compared to the native lung. Methionine and cysteine metabolism pathways are known to regulate oxidative stress through the methionine/glutathinone trans-sulfuration pathway. Disruption of this pathway can lead to increased oxidative injury. Likewise, increased fatty acid activation can result in mobilization of cell membrane derived lipid signaling molecules such as sphingospine-1-kinase and arachidonic acid derived eicosainoides [57, 58]. More studies are needed to further understand if the variations in metabolome are in part influenced by the difference in airway microbiome or vice-versa.

As the primary antigen presenting cells of the lung, resident alveolar macrophages originate in the embryogenesis period and are sparsely replenished by the bone marrow during adult life [59]. Studies in lung transplant recipients have shown that most of the alveolar macrophages even 3.5 years after lung transplant are donor derived in the allografts [60]. An alternate explanation to the difference in the lung microbiome in the native and allograft lungs could be due to the variable antigen presenting/regulatory nature of donor-derived and recipient alveolar macrophage phenotypes in the allografts and native lungs.

Our study has limitations, including sample size and design. We did not find any differences in the microbiome or inflammatory signatures in those diagnosed with CLAD at sampling or at follow-up. However, with a cross-sectional study design and inadequate power, we cannot comment on the dynamic changes in the microbiome and metabolome of native lungs and allografts and their association with chronic lung allograft dysfunction. However, this study is hypothesis generating and provides insightful data to further advance our understanding of microbiome-metabolome interactions in the lung. One of the unique challenges in the field of lung microbiome is presence of low biomass in BAL samples compared to samples from the GI tract [61, 62]. Interpretation of results obtained from a single low biomass sample without appropriate controls can be misleading. To circumvent this issue, we collected control samples from the separate bronchoscopes used during each A and N sample collection. The bronchoscope control microbiome signatures were found to be dissimilar to the A and N airway microbiome (Figure S2A, B). Furthermore, the bacterial 16S DNA levels in the bronchoscope control samples were below the lower limit of PCR quantification suggesting absence of bronchoscope contamination (data not shown). BAL samples from normal lung control subjects, collected and processed using a similar methodology were also analyzed and compared to the allograft/native lung microbiome. We processed technical replicates of each biological sample to evaluate variability in both the relative abundance and bacterial 16S DNA quantification. Although, antimicrobial prophylaxis and immunosuppression regimen can also influence the microbiome [63], in our subjects these were given systemically, and hence would impact both the native and the allograft similarly. While we found that lung allografts harbored a more *Proteobacteria* dominant microbiome and higher PGP levels, longitudinal studies are needed to investigate the causal association, role of varied prophylactic antibiotics, immunosuppression on microbial composition in the lung and whether this inflammation translates into allograft rejection. Our healthy controls did not have Ac-PGP measurements. Nevertheless, our previous studies have shown absent or extremely low levels of Ac-PGP in healthy lung airway fluid [64]. It has been reported that BAL samples obtained from separate geographic regions within the same lung can demonstrate highly dissimilar microbial communities [33, 65]. Although, it is possible that the variations in microbiome in the native lung and allograft may be related to expected variation in sampling from different regions of the lung, these spatial variations are known to be less significant compared to variations across individuals [65]. Finally, all metabolites that correlated with bacterial genera were not able to be identified due to the current limitations of untargeted mass spectrometry and the adjusted *P* values had limitations due to the smaller samples size [66]. Although, we found correlations between sphingosine like molecules with bacterial genera in the native lungs, due to the very low concentration of these sphingosine metabolites in the allograft, these bacterial-sphingosine correlations could not be accurately predicted in the allograft and targeted LC-MS analysis was not performed to validate the metabolite. Nevertheless, these observations are hypothesis generating and warrant further validation in future mechanistic studies.

## Conclusion

Allograft lungs have a distinct microbiome signature, and an increased pro-inflammatory milieu compared to the native lungs in SLTs. Airway metabolome distinguishes the allografts from native lungs and is associated with distinct microbial communities, suggesting a functional relationship between the local microbiome and metabolome. Our studies have characterized, for the first time, the native lung and allograft microbiome of SLTs and provide fundamental insight into host-microbiome and metabolome interactions, a potentially important early feature for long-term allograft viability and host-pathogen related lung injury. These hypothesis generating results pave way for future well powered longitudinal studies to elucidate dynamic changes in the lung microbiome and metabolome, determine their associated interactions, and measure their subsequent impact on lung allograft health and function.

## Supplementary information

**Additional file 1.**

**Additional file 2.**

**Additional file 3.**

**Additional file 4.**

**Additional file 5.**

**Additional file 6.**

**Additional file 7.**

**Additional file 8.**

**Additional file 9.**

## Data Availability

The data that support the findings of this study are openly available in UAB CCTS repository at https://data.genome.uab.edu/bmi/microbiome/result2017/M146_analysis/Sharma2nd_analysis/ANALYSIS/report_files/initial_overview.html

## Abbreviations

BAL: Bronchoalveolar lavage
CLAD: Chronic Lung Allograft Dysfunction
SLTs: Single lung transplant recipients
