# Supplementary material for: Differences in airway microbiome and metabolome of single lung transplant recipients
Source: Respir Res. 2020 May 6;21:104. doi: 10.1186/s12931-020-01367-3 (PMC7201609; doi:10.1186/s12931-020-01367-3)
Supplement: Supplementary file 2 — Additional file 2. [file 12931_2020_1367_MOESM2_ESM.docx]

| Consensus Lineage | P Value | FDR P value |
| --- | --- | --- |
| k__Bacteria; p__Proteobacteria; c__Epsilonproteobacteria; o__Campylobacterales; f__Helicobacteraceae | 4.2E-07 | 8.61E-05 |
| k__Bacteria; p__Firmicutes; c__Clostridia; o__Clostridiales | 4.41E-07 | 4.52E-05 |
| k__Bacteria; p__Proteobacteria; c__Betaproteobacteria; o__Burkholderiales; f__Alcaligenaceae; g__Sutterella; s__ | 7.33E-07 | 5.01E-05 |
| k__Bacteria; p__Firmicutes; c__Clostridia; o__Clostridiales; f__[Tissierellaceae]; g__Peptoniphilus; s__ | 1.9E-06 | 9.74E-05 |
| k__Bacteria; p__Firmicutes; c__Clostridia; o__Clostridiales | 2.12E-06 | 8.70E-05 |
| k__Bacteria; p__Bacteroidetes; c__Bacteroidia; o__Bacteroidales; f__S24-7; g__; s__ | 3.42E-06 | 0.0001167 |
| k__Bacteria; p__Bacteroidetes; c__Bacteroidia; o__Bacteroidales; f__S24-7; g__; s__ | 5.45E-06 | 0.0001595 |
| k__Bacteria; p__Firmicutes; c__Bacilli; o__Turicibacterales; f__Turicibacteraceae; g__Turicibacter; s__ | 6.83E-06 | 0.0001751 |
| k__Bacteria; p__Bacteroidetes; c__Bacteroidia; o__Bacteroidales; f__[Paraprevotellaceae]; g__[Prevotella]; s__ | 8.58E-06 | 0.0001955 |
| k__Bacteria; p__Proteobacteria; c__Betaproteobacteria; o__Burkholderiales; f__Alcaligenaceae; g__Sutterella; s__ | 1.49E-05 | 0.0003059 |
| k__Bacteria; p__Bacteroidetes; c__Bacteroidia; o__Bacteroidales; f__S24-7; g__; s__ | 1.94E-05 | 0.0003617 |
| k__Bacteria; p__Firmicutes; c__Clostridia; o__Clostridiales; f__Ruminococcaceae; g__Oscillospira; s__ | 2.17E-05 | 0.0003699 |
| k__Bacteria; p__Proteobacteria; c__Betaproteobacteria; o__Burkholderiales; f__Alcaligenaceae; g__Sutterella; s__ | 2.73E-05 | 0.0004313 |
| k__Bacteria; p__Bacteroidetes; c__Bacteroidia; o__Bacteroidales; f__[Odoribacteraceae]; g__Odoribacter; s__ | 2.79E-05 | 0.000409 |
| k__Bacteria; p__Bacteroidetes; c__Bacteroidia; o__Bacteroidales; f__Bacteroidaceae; g__Bacteroides | 4.47E-05 | 0.0006113 |
| k__Bacteria; p__Bacteroidetes; c__Bacteroidia; o__Bacteroidales; f__Prevotellaceae; g__Prevotella; s__ | 6.45E-05 | 0.0008263 |
| k__Bacteria; p__Bacteroidetes; c__Bacteroidia; o__Bacteroidales; f__Bacteroidaceae; g__Bacteroides | 7.29E-05 | 0.0008789 |
| k__Bacteria; p__Firmicutes; c__Clostridia; o__Clostridiales; f__Lachnospiraceae; g__Coprococcus; s__ | 7.53E-05 | 0.000858 |
| k__Bacteria; p__Proteobacteria; c__Epsilonproteobacteria; o__Campylobacterales; f__Helicobacteraceae; g__Sulfurimonas; s__ | 0.000104 | 0.0011209 |
| k__Bacteria; p__Proteobacteria; c__Deltaproteobacteria; o__Desulfovibrionales; f__Desulfovibrionaceae; g__; s__ | 0.000111 | 0.0011421 |
| k__Bacteria; p__Proteobacteria; c__Deltaproteobacteria; o__Desulfovibrionales; f__Desulfovibrionaceae; g__Desulfovibrio; s__C21_c20 | 0.000149 | 0.0014529 |
| k__Bacteria; p__Firmicutes; c__Clostridia; o__Clostridiales; f__Veillonellaceae; g__Veillonella; s__ | 0.000158 | 0.0014757 |
| k__Bacteria; p__Bacteroidetes; c__Bacteroidia; o__Bacteroidales; f__S24-7; g__; s__ | 0.000169 | 0.0015103 |
| k__Bacteria; p__Firmicutes; c__Clostridia; o__Clostridiales; f__Veillonellaceae; g__Veillonella; s__ | 0.00017 | 0.0014539 |
| k__Bacteria; p__Proteobacteria; c__Deltaproteobacteria; o__Desulfovibrionales; f__Desulfovibrionaceae; g__Bilophila; s__ | 0.000223 | 0.0018295 |
| k__Bacteria; p__Bacteroidetes; c__Bacteroidia; o__Bacteroidales; f__S24-7; g__; s__ | 0.000244 | 0.0019205 |
| k__Bacteria; p__Firmicutes; c__Clostridia; o__Clostridiales | 0.000244 | 0.0018536 |
| k__Bacteria; p__Bacteroidetes; c__Bacteroidia; o__Bacteroidales; f__S24-7; g__; s__ | 0.00027 | 0.0019781 |
| k__Bacteria; p__Firmicutes; c__Clostridia; o__Clostridiales; f__Lachnospiraceae | 0.000297 | 0.0021022 |
| k__Bacteria; p__Proteobacteria; c__Gammaproteobacteria | 0.000299 | 0.002045 |
| k__Bacteria; p__Firmicutes; c__Clostridia; o__Clostridiales; f__Lachnospiraceae; g__Dorea; s__ | 0.000374 | 0.0024762 |
| k__Bacteria; p__Firmicutes; c__Clostridia; o__Clostridiales | 0.000437 | 0.0027967 |
| k__Bacteria; p__Bacteroidetes; c__Flavobacteriia; o__Flavobacteriales; f__; g__; s__ | 0.000535 | 0.0033239 |
| k__Bacteria; p__Bacteroidetes; c__Bacteroidia; o__Bacteroidales; f__Bacteroidaceae; g__Bacteroides | 0.000542 | 0.0032691 |
| k__Bacteria; p__Firmicutes; c__Clostridia; o__Clostridiales; f__Dehalobacteriaceae; g__Dehalobacterium; s__ | 0.000562 | 0.0032928 |
| k__Bacteria; p__Firmicutes; c__Clostridia; o__Clostridiales | 0.000658 | 0.0037494 |
| k__Bacteria; p__Firmicutes; c__Erysipelotrichi; o__Erysipelotrichales; f__Erysipelotrichaceae | 0.000684 | 0.003789 |
| k__Bacteria; p__Firmicutes; c__Bacilli; o__Lactobacillales; f__Lactobacillaceae; g__Lactobacillus; s__ | 0.000695 | 0.0037499 |
| k__Bacteria; p__Bacteroidetes; c__Bacteroidia; o__Bacteroidales; f__Bacteroidaceae; g__Bacteroides; s__caccae | 0.000741 | 0.0038928 |
| k__Bacteria; p__Firmicutes; c__Clostridia; o__Clostridiales; f__Lachnospiraceae | 0.000752 | 0.0038524 |
| k__Bacteria; p__Proteobacteria; c__Gammaproteobacteria; o__Alteromonadales; f__Colwelliaceae; g__; s__ | 0.000759 | 0.0037974 |
| k__Bacteria; p__Actinobacteria; c__Actinobacteria; o__Actinomycetales; f__Corynebacteriaceae; g__Corynebacterium | 0.000782 | 0.0038149 |
| k__Bacteria; p__Bacteroidetes; c__Bacteroidia; o__Bacteroidales; f__Porphyromonadaceae; g__Porphyromonas; s__ | 0.000814 | 0.0038814 |
| k__Bacteria; p__Bacteroidetes; c__Bacteroidia; o__Bacteroidales; f__S24-7; g__; s__ | 0.000918 | 0.0042754 |
| k__Bacteria; p__Bacteroidetes; c__Bacteroidia; o__Bacteroidales; f__; g__; s__ | 0.001085 | 0.0049429 |
| k__Bacteria; p__Bacteroidetes; c__Bacteroidia; o__Bacteroidales; f__Bacteroidaceae; g__Bacteroides; s__ovatus | 0.001132 | 0.0050447 |
| k__Bacteria; p__Firmicutes; c__Clostridia; o__Clostridiales; f__Lachnospiraceae | 0.001149 | 0.0050126 |
| k__Bacteria; p__Firmicutes; c__Clostridia; o__Clostridiales; f__Lachnospiraceae | 0.001261 | 0.0053865 |
| k__Bacteria; p__Bacteroidetes; c__Bacteroidia; o__Bacteroidales; f__Bacteroidaceae; g__Bacteroides; s__acidifaciens | 0.001366 | 0.0057128 |
| k__Bacteria; p__Proteobacteria; c__Gammaproteobacteria; o__Alteromonadales; f__Colwelliaceae; g__; s__ | 0.001374 | 0.0056337 |
| k__Bacteria; p__Actinobacteria; c__Actinobacteria; o__Bifidobacteriales; f__Bifidobacteriaceae | 0.001375 | 0.005525 |
| k__Bacteria; p__Firmicutes; c__Bacilli; o__Lactobacillales; f__Lactobacillaceae; g__Lactobacillus; s__ | 0.001401 | 0.0055244 |
| k__Bacteria; p__Actinobacteria; c__Actinobacteria; o__Actinomycetales; f__Micrococcaceae; g__Sinomonas; s__ | 0.001442 | 0.0055786 |
| k__Bacteria; p__Bacteroidetes; c__Flavobacteriia; o__Flavobacteriales; f__[Weeksellaceae]; g__Chryseobacterium; s__ | 0.001537 | 0.0058339 |
| k__Bacteria; p__Firmicutes; c__Erysipelotrichi; o__Erysipelotrichales; f__Erysipelotrichaceae; g__Allobaculum; s__ | 0.00157 | 0.0058508 |
| k__Bacteria; p__Bacteroidetes; c__Bacteroidia; o__Bacteroidales; f__[Paraprevotellaceae]; g__[Prevotella]; s__ | 0.001781 | 0.0065182 |
| k__Bacteria; p__Tenericutes; c__Mollicutes; o__Mycoplasmatales; f__Mycoplasmataceae; g__Mycoplasma; s__genitalium | 0.001831 | 0.0065844 |
| k__Bacteria; p__Tenericutes; c__Mollicutes; o__Mycoplasmatales; f__Mycoplasmataceae; g__; s__ | 0.001875 | 0.0066256 |
| k__Bacteria; p__Bacteroidetes; c__Bacteroidia; o__Bacteroidales | 0.00188 | 0.0065322 |
| k__Bacteria; p__Bacteroidetes; c__Bacteroidia; o__Bacteroidales; f__S24-7; g__; s__ | 0.00189 | 0.0064563 |
| k__Bacteria; p__Bacteroidetes; c__Bacteroidia; o__Bacteroidales; f__Bacteroidaceae; g__Bacteroides; s__ | 0.001927 | 0.0064773 |
| k__Bacteria; p__Bacteroidetes; c__Bacteroidia; o__Bacteroidales; f__Prevotellaceae; g__Prevotella; s__ | 0.002063 | 0.0068226 |
| k__Bacteria; p__Bacteroidetes; c__Bacteroidia; o__Bacteroidales; f__Rikenellaceae; g__; s__ | 0.002244 | 0.0073013 |
| k__Bacteria; p__Bacteroidetes; c__Bacteroidia; o__Bacteroidales; f__Bacteroidaceae; g__Bacteroides; s__ | 0.00247 | 0.0079123 |
| k__Bacteria; p__Bacteroidetes; c__Bacteroidia; o__Bacteroidales; f__Prevotellaceae; g__Prevotella; s__ | 0.002665 | 0.008406 |
| k__Bacteria; p__Firmicutes; c__Erysipelotrichi; o__Erysipelotrichales; f__Erysipelotrichaceae; g__[Eubacterium]; s__dolichum | 0.002669 | 0.0082887 |
| k__Bacteria; p__Bacteroidetes; c__Bacteroidia; o__Bacteroidales; f__S24-7; g__; s__ | 0.002711 | 0.0082958 |
| k__Bacteria; p__Proteobacteria; c__Betaproteobacteria; o__Neisseriales; f__Neisseriaceae; g__Neisseria | 0.002836 | 0.0085504 |
| k__Bacteria; p__Firmicutes; c__Clostridia; o__Clostridiales | 0.002939 | 0.0087309 |
| k__Bacteria; p__Bacteroidetes; c__Bacteroidia; o__Bacteroidales; f__Bacteroidaceae; g__Bacteroides; s__ | 0.002989 | 0.0087527 |
| k__Bacteria; p__Bacteroidetes | 0.003071 | 0.0088669 |
| k__Bacteria; p__Firmicutes; c__Clostridia; o__Clostridiales; f__Lachnospiraceae; g__; s__ | 0.003286 | 0.0093561 |
| k__Bacteria; p__Firmicutes; c__Bacilli; o__Lactobacillales; f__Lactobacillaceae; g__Lactobacillus; s__ | 0.003302 | 0.0092715 |
| k__Bacteria; p__Bacteroidetes; c__Bacteroidia; o__Bacteroidales; f__S24-7; g__; s__ | 0.003448 | 0.009553 |
| k__Bacteria; p__Firmicutes; c__Clostridia; o__Clostridiales; f__Lachnospiraceae; g__; s__ | 0.003575 | 0.009771 |
| k__Bacteria; p__Bacteroidetes; c__Bacteroidia; o__Bacteroidales; f__Porphyromonadaceae; g__Porphyromonas; s__ | 0.003662 | 0.0098766 |
| k__Bacteria; p__Firmicutes; c__Bacilli; o__Bacillales; f__Bacillaceae | 0.004262 | 0.0113474 |
| k__Bacteria; p__Bacteroidetes; c__Bacteroidia; o__Bacteroidales; f__S24-7; g__; s__ | 0.004325 | 0.011368 |
| k__Bacteria; p__Firmicutes; c__Clostridia; o__Clostridiales; f__Veillonellaceae; g__Megasphaera; s__ | 0.004368 | 0.0113341 |
| k__Bacteria; p__Bacteroidetes; c__Bacteroidia; o__Bacteroidales; f__Rikenellaceae; g__AF12; s__ | 0.004503 | 0.0115378 |
| k__Bacteria; p__Bacteroidetes; c__Bacteroidia; o__Bacteroidales; f__Rikenellaceae; g__; s__ | 0.004536 | 0.0114802 |
| k__Bacteria; p__Firmicutes; c__Clostridia; o__Clostridiales; f__[Tissierellaceae]; g__WAL_1855D; s__ | 0.004624 | 0.0115603 |
| k__Bacteria; p__Firmicutes; c__Bacilli; o__Bacillales; f__Alicyclobacillaceae; g__Alicyclobacillus; s__ | 0.004747 | 0.0117234 |
| k__Bacteria; p__Proteobacteria; c__Gammaproteobacteria; o__Alteromonadales; f__Psychromonadaceae; g__Psychromonas; s__ | 0.005712 | 0.0139393 |
| k__Bacteria; p__Firmicutes; c__Clostridia; o__Clostridiales; f__[Tissierellaceae]; g__1-68; s__ | 0.006011 | 0.0144977 |
| k__Bacteria; p__Firmicutes; c__Clostridia; o__Clostridiales; f__[Tissierellaceae]; g__ph2; s__ | 0.006305 | 0.0150305 |
| k__Bacteria; p__Fusobacteria; c__Fusobacteriia; o__Fusobacteriales; f__Fusobacteriaceae; g__Propionigenium; s__ | 0.006324 | 0.0149011 |
| k__Bacteria; p__Bacteroidetes; c__Bacteroidia; o__Bacteroidales; f__S24-7; g__; s__ | 0.006364 | 0.0148255 |
| k__Bacteria; p__Verrucomicrobia; c__Verrucomicrobiae; o__Verrucomicrobiales; f__Verrucomicrobiaceae; g__Akkermansia; s__muciniphila | 0.006664 | 0.0153494 |
| k__Bacteria; p__Bacteroidetes; c__Bacteroidia; o__Bacteroidales; f__S24-7; g__; s__ | 0.006968 | 0.015871 |
| k__Bacteria; p__Verrucomicrobia; c__Verrucomicrobiae; o__Verrucomicrobiales; f__Verrucomicrobiaceae; g__Akkermansia; s__muciniphila | 0.007027 | 0.0158304 |
| k__Bacteria; p__Tenericutes; c__Mollicutes; o__Anaeroplasmatales; f__Anaeroplasmataceae; g__Anaeroplasma; s__ | 0.007055 | 0.0157196 |
| k__Bacteria; p__Bacteroidetes; c__Bacteroidia; o__Bacteroidales; f__S24-7; g__; s__ | 0.00711 | 0.0156718 |
| k__Bacteria; p__Firmicutes; c__Clostridia; o__Clostridiales; f__Clostridiaceae; g__SMB53; s__ | 0.007293 | 0.0159059 |
| k__Bacteria; p__Bacteroidetes; c__Bacteroidia; o__Bacteroidales; f__Rikenellaceae; g__Rikenella; s__ | 0.007564 | 0.0163229 |
| k__Bacteria; p__Bacteroidetes; c__Bacteroidia; o__Bacteroidales; f__Bacteroidaceae; g__Bacteroides | 0.007686 | 0.0164132 |
| k__Bacteria; p__Firmicutes; c__Clostridia; o__Clostridiales; f__Ruminococcaceae; g__Anaerotruncus; s__ | 0.008014 | 0.0169363 |
| k__Bacteria; p__Actinobacteria; c__Actinobacteria; o__Actinomycetales; f__Mycobacteriaceae; g__Mycobacterium; s__ | 0.008364 | 0.0174951 |
| k__Bacteria; p__Proteobacteria; c__Deltaproteobacteria; o__Desulfobacterales; f__Desulfobulbaceae; g__Desulfotalea; s__ | 0.008522 | 0.0176476 |
| k__Bacteria; p__Proteobacteria; c__Alphaproteobacteria; o__Sphingomonadales; f__Sphingomonadaceae; g__Sphingomonas | 0.009107 | 0.018669 |
| k__Bacteria; p__Bacteroidetes; c__Flavobacteriia; o__Flavobacteriales; f__; g__; s__ | 0.009163 | 0.0185987 |
| k__Bacteria; p__Bacteroidetes; c__Bacteroidia; o__Bacteroidales; f__Bacteroidaceae; g__Bacteroides | 0.009555 | 0.0192033 |
| k__Bacteria | 0.009942 | 0.019788 |
| k__Bacteria; p__Proteobacteria; c__Gammaproteobacteria; o__Alteromonadales; f__Colwelliaceae; g__Thalassomonas; s__ | 0.010053 | 0.0198159 |
| k__Bacteria; p__Firmicutes; c__Bacilli; o__Lactobacillales; f__Lactobacillaceae; g__Lactobacillus; s__ | 0.010227 | 0.0199664 |
| k__Bacteria; p__Firmicutes; c__Clostridia; o__Clostridiales; f__[Tissierellaceae]; g__Anaerococcus; s__ | 0.010274 | 0.0198705 |
| k__Bacteria; p__Firmicutes; c__Clostridia; o__Clostridiales; f__Lachnospiraceae; g__; s__ | 0.010315 | 0.0197623 |
| k__Bacteria; p__Firmicutes; c__Clostridia; o__Clostridiales; f__Lachnospiraceae; g__; s__ | 0.010346 | 0.0196373 |
| k__Bacteria; p__Bacteroidetes; c__Bacteroidia; o__Bacteroidales; f__Porphyromonadaceae; g__Parabacteroides; s__ | 0.010643 | 0.0200158 |
| k__Bacteria; p__Proteobacteria; c__Gammaproteobacteria; o__Vibrionales; f__Vibrionaceae; g__Vibrio; s__rumoiensis | 0.010663 | 0.0198714 |
| k__Bacteria; p__Firmicutes; c__Clostridia; o__Clostridiales; f__; g__; s__ | 0.01067 | 0.0197065 |
| k__Bacteria; p__Fusobacteria; c__Fusobacteriia; o__Fusobacteriales; f__Leptotrichiaceae; g__Sneathia; s__ | 0.011382 | 0.0208332 |
| k__Bacteria; p__Actinobacteria; c__Actinobacteria; o__Bifidobacteriales; f__Bifidobacteriaceae; g__Bifidobacterium | 0.011683 | 0.0211949 |
| k__Bacteria; p__Bacteroidetes; c__Bacteroidia; o__Bacteroidales; f__Prevotellaceae; g__Prevotella; s__ | 0.011684 | 0.0210109 |
| k__Bacteria; p__Proteobacteria; c__Gammaproteobacteria; o__Vibrionales; f__Vibrionaceae | 0.011892 | 0.0211991 |
| k__Bacteria; p__Firmicutes; c__Clostridia; o__Clostridiales; f__Lachnospiraceae; g__; s__ | 0.012099 | 0.0213815 |
| k__Bacteria; p__Firmicutes; c__Bacilli; o__Bacillales; f__Bacillaceae | 0.012325 | 0.0215957 |
| k__Bacteria; p__Tenericutes; c__Mollicutes; o__Mycoplasmatales; f__Mycoplasmataceae; g__Mycoplasma; s__ | 0.012407 | 0.0215553 |
| k__Bacteria; p__Proteobacteria; c__Gammaproteobacteria; o__Alteromonadales; f__Psychromonadaceae; g__Psychromonas; s__ | 0.012596 | 0.021699 |
| k__Bacteria; p__Firmicutes; c__Clostridia; o__Clostridiales; f__; g__; s__ | 0.012623 | 0.0215636 |
| k__Bacteria; p__Actinobacteria; c__Actinobacteria; o__Actinomycetales; f__Corynebacteriaceae; g__Corynebacterium; s__ | 0.01272 | 0.0215505 |
| k__Bacteria; p__Bacteroidetes; c__Bacteroidia; o__Bacteroidales; f__S24-7; g__; s__ | 0.013384 | 0.0224901 |
| k__Bacteria; p__Bacteroidetes; c__Bacteroidia; o__Bacteroidales; f__S24-7; g__; s__ | 0.013975 | 0.023292 |
| k__Bacteria; p__Firmicutes; c__Bacilli; o__Lactobacillales; f__Aerococcaceae; g__Aerococcus; s__ | 0.01435 | 0.0237235 |
| k__Bacteria; p__Actinobacteria; c__Actinobacteria; o__Bifidobacteriales; f__Bifidobacteriaceae; g__Bifidobacterium | 0.014545 | 0.0238545 |
| k__Bacteria; p__Cyanobacteria; c__Chloroplast; o__Rhodophyta; f__; g__; s__ | 0.014576 | 0.0237146 |
| k__Bacteria; p__Firmicutes; c__Clostridia; o__Clostridiales; f__Ruminococcaceae; g__Oscillospira; s__ | 0.015133 | 0.0244279 |
| k__Bacteria; p__Bacteroidetes; c__Bacteroidia; o__Bacteroidales; f__Prevotellaceae; g__Prevotella; s__ | 0.015375 | 0.0246243 |
| k__Bacteria; p__Bacteroidetes; c__Flavobacteriia; o__Flavobacteriales; f__[Weeksellaceae]; g__Cloacibacterium; s__ | 0.016482 | 0.0261925 |
| k__Bacteria; p__Firmicutes; c__Clostridia; o__Clostridiales; f__Ruminococcaceae; g__Oscillospira; s__ | 0.016561 | 0.0261149 |
| k__Bacteria; p__Firmicutes; c__Clostridia; o__Clostridiales; f__Lachnospiraceae | 0.016921 | 0.026479 |
| k__Bacteria; p__Proteobacteria; c__Gammaproteobacteria; o__Alteromonadales; f__Psychromonadaceae; g__Psychromonas; s__ | 0.017596 | 0.0273265 |
| k__Bacteria; p__Proteobacteria; c__Gammaproteobacteria; o__Thiohalorhabdales; f__; g__; s__ | 0.01787 | 0.0275434 |
| k__Bacteria; p__Firmicutes; c__Clostridia; o__Clostridiales | 0.018237 | 0.0279 |
| k__Bacteria; p__Firmicutes; c__Erysipelotrichi; o__Erysipelotrichales; f__Erysipelotrichaceae; g__Allobaculum; s__ | 0.019061 | 0.0289444 |
| k__Bacteria; p__Bacteroidetes; c__Bacteroidia; o__Bacteroidales; f__Bacteroidaceae; g__Bacteroides; s__ | 0.019256 | 0.0290256 |
| k__Bacteria; p__Tenericutes; c__Mollicutes; o__Mycoplasmatales; f__Mycoplasmataceae; g__Ureaplasma; s__ | 0.019555 | 0.0292617 |
| k__Bacteria; p__Firmicutes; c__Clostridia; o__Clostridiales; f__Lachnospiraceae; g__Shuttleworthia; s__ | 0.01981 | 0.029428 |
| k__Bacteria; p__Proteobacteria; c__Gammaproteobacteria; o__Pseudomonadales; f__Pseudomonadaceae; g__Pseudomonas | 0.01983 | 0.0292454 |
| k__Bacteria; p__Actinobacteria; c__Actinobacteria; o__Actinomycetales; f__Corynebacteriaceae; g__Corynebacterium; s__ | 0.019957 | 0.0292225 |
| k__Bacteria; p__Proteobacteria; c__Gammaproteobacteria; o__Pseudomonadales; f__Pseudomonadaceae | 0.020431 | 0.0297049 |
| k__Bacteria; p__Proteobacteria; c__Gammaproteobacteria; o__Pseudomonadales; f__Pseudomonadaceae; g__Pseudomonas; s__fragi | 0.020719 | 0.0299119 |
| k__Bacteria; p__Firmicutes; c__Clostridia; o__Clostridiales; f__Ruminococcaceae; g__Oscillospira; s__ | 0.022503 | 0.032259 |
| k__Bacteria; p__Actinobacteria; c__Actinobacteria; o__Bifidobacteriales; f__Bifidobacteriaceae | 0.022547 | 0.0320981 |
| k__Bacteria; p__Firmicutes | 0.023003 | 0.0325221 |
| k__Bacteria; p__Bacteroidetes; c__Bacteroidia; o__Bacteroidales; f__S24-7; g__; s__ | 0.023243 | 0.0326353 |
| k__Bacteria; p__Firmicutes; c__Clostridia; o__Clostridiales; f__Lachnospiraceae; g__; s__ | 0.023829 | 0.0332304 |
| k__Bacteria | 0.024005 | 0.0332495 |
| k__Bacteria; p__Proteobacteria; c__Epsilonproteobacteria; o__Campylobacterales; f__Helicobacteraceae; g__Helicobacter; s__ | 0.025006 | 0.0344038 |
| k__Bacteria; p__Firmicutes; c__Bacilli; o__Lactobacillales; f__Lactobacillaceae; g__Lactobacillus; s__ | 0.02521 | 0.0344539 |
| k__Bacteria; p__Actinobacteria; c__Acidimicrobiia; o__Acidimicrobiales; f__JdFBGBact; g__; s__ | 0.025355 | 0.0344226 |
| k__Bacteria; p__Actinobacteria; c__Actinobacteria; o__Bifidobacteriales; f__Bifidobacteriaceae; g__Bifidobacterium | 0.02595 | 0.0349978 |
| k__Bacteria; p__Actinobacteria; c__Coriobacteriia; o__Coriobacteriales; f__Coriobacteriaceae; g__Atopobium; s__vaginae | 0.02662 | 0.0356675 |
| k__Bacteria; p__Bacteroidetes; c__Bacteroidia; o__Bacteroidales; f__Prevotellaceae; g__Prevotella | 0.026886 | 0.03579 |
| k__Bacteria; p__Proteobacteria; c__Gammaproteobacteria; o__Alteromonadales; f__Moritellaceae; g__Moritella; s__ | 0.027382 | 0.0362147 |
| k__Bacteria; p__Proteobacteria; c__Betaproteobacteria; o__Burkholderiales; f__Oxalobacteraceae | 0.027474 | 0.0361037 |
| k__Bacteria; p__Proteobacteria; c__Betaproteobacteria; o__Rhodocyclales; f__Rhodocyclaceae; g__Hydrogenophilus; s__ | 0.028029 | 0.0365978 |
| k__Bacteria; p__Firmicutes; c__Clostridia; o__Clostridiales; f__Clostridiaceae | 0.029337 | 0.0380639 |
| k__Bacteria; p__Proteobacteria; c__Gammaproteobacteria; o__Thiotrichales; f__Thiotrichaceae; g__Cocleimonas; s__ | 0.029905 | 0.0385569 |
| k__Bacteria; p__Firmicutes; c__Bacilli; o__Lactobacillales; f__Lactobacillaceae; g__Lactobacillus; s__iners | 0.030517 | 0.0391001 |
| k__Bacteria; p__Bacteroidetes; c__Bacteroidia; o__Bacteroidales; f__Bacteroidaceae; g__Bacteroides | 0.030774 | 0.0391841 |
| k__Bacteria; p__Firmicutes; c__Clostridia; o__Clostridiales; f__[Tissierellaceae]; g__Peptoniphilus; s__ | 0.031059 | 0.0393029 |
| k__Bacteria; p__Bacteroidetes; c__Bacteroidia; o__Bacteroidales; f__Prevotellaceae; g__Prevotella; s__ | 0.031164 | 0.0391937 |
| k__Bacteria; p__Firmicutes; c__Clostridia; o__Clostridiales; f__Ruminococcaceae; g__Oscillospira; s__ | 0.0316 | 0.0395005 |
| k__Bacteria; p__Firmicutes; c__Clostridia; o__Clostridiales; f__; g__; s__ | 0.031811 | 0.0395226 |
| k__Bacteria; p__Firmicutes; c__Bacilli; o__Lactobacillales; f__Lactobacillaceae; g__Lactobacillus; s__iners | 0.032085 | 0.0396227 |
| k__Bacteria | 0.032148 | 0.0394631 |
| k__Bacteria; p__Bacteroidetes; c__Flavobacteriia; o__Flavobacteriales; f__Flavobacteriaceae | 0.032289 | 0.0393997 |
| k__Bacteria; p__Firmicutes; c__Clostridia; o__Clostridiales; f__Lachnospiraceae | 0.033036 | 0.0400735 |
| k__Bacteria; p__Firmicutes; c__Bacilli; o__Lactobacillales; f__Lactobacillaceae; g__Lactobacillus; s__ | 0.033909 | 0.0408902 |
| k__Bacteria; p__Firmicutes; c__Clostridia; o__Clostridiales; f__Veillonellaceae; g__Dialister; s__ | 0.034061 | 0.0408338 |
| k__Bacteria; p__Proteobacteria; c__Gammaproteobacteria; o__Xanthomonadales; f__Xanthomonadaceae; g__Stenotrophomonas; s__ | 0.034592 | 0.0412291 |
| k__Bacteria; p__Bacteroidetes; c__Bacteroidia; o__Bacteroidales; f__S24-7; g__; s__ | 0.035634 | 0.0422249 |
| k__Bacteria; p__Firmicutes; c__Clostridia; o__Clostridiales | 0.035739 | 0.0421062 |
| k__Bacteria; p__Firmicutes; c__Clostridia; o__Clostridiales; f__Lachnospiraceae; g__; s__ | 0.035796 | 0.0419328 |
| k__Bacteria; p__Bacteroidetes; c__Bacteroidia; o__Bacteroidales; f__Porphyromonadaceae; g__Parabacteroides; s__ | 0.037789 | 0.0440151 |
| k__Bacteria; p__Proteobacteria; c__Epsilonproteobacteria; o__Campylobacterales; f__Campylobacteraceae; g__Arcobacter; s__ | 0.038348 | 0.0444141 |
| k__Bacteria; p__Firmicutes; c__Clostridia; o__Clostridiales; f__Lachnospiraceae; g__Coprococcus; s__ | 0.03868 | 0.0445476 |
| k__Bacteria; p__Bacteroidetes; c__Bacteroidia; o__Bacteroidales; f__Prevotellaceae; g__Prevotella; s__ | 0.040279 | 0.0461295 |
| k__Bacteria; p__Proteobacteria; c__Gammaproteobacteria; o__Alteromonadales; f__Colwelliaceae; g__Thalassomonas | 0.040474 | 0.0460956 |
| k__Bacteria; p__Bacteroidetes; c__Bacteroidia; o__Bacteroidales; f__Bacteroidaceae; g__Bacteroides | 0.040825 | 0.0462384 |
| k__Bacteria; p__Firmicutes; c__Bacilli; o__Bacillales; f__Bacillaceae; g__Bacillus; s__cereus | 0.040932 | 0.0461043 |
| k__Bacteria; p__Firmicutes; c__Clostridia; o__Clostridiales | 0.041099 | 0.0460402 |
| k__Bacteria; p__Firmicutes; c__Clostridia; o__Clostridiales; f__Lachnospiraceae | 0.041132 | 0.0458268 |
| k__Bacteria; p__Tenericutes; c__Mollicutes; o__Mycoplasmatales; f__Mycoplasmataceae; g__; s__ | 0.042134 | 0.0466888 |
| k__Bacteria; p__Firmicutes; c__Clostridia; o__Clostridiales; f__Ruminococcaceae | 0.042511 | 0.0468537 |
| k__Bacteria; p__Firmicutes; c__Bacilli; o__Lactobacillales; f__Lactobacillaceae; g__Lactobacillus | 0.044495 | 0.0487783 |
| k__Bacteria; p__Firmicutes; c__Clostridia; o__Clostridiales; f__Ruminococcaceae; g__Ruminococcus; s__ | 0.044548 | 0.0485762 |
| k__Bacteria; p__Proteobacteria; c__Gammaproteobacteria; o__; f__; g__; s__ | 0.044684 | 0.0484672 |
| k__Bacteria; p__Firmicutes; c__Clostridia; o__Clostridiales | 0.044728 | 0.0482593 |
| k__Bacteria; p__Proteobacteria; c__Gammaproteobacteria; o__Vibrionales; f__Vibrionaceae | 0.045772 | 0.0491274 |
| k__Bacteria; p__Firmicutes; c__Bacilli; o__Lactobacillales; f__Lactobacillaceae; g__Lactobacillus | 0.046305 | 0.0494401 |
| k__Bacteria; p__Actinobacteria; c__Actinobacteria; o__Actinomycetales; f__Micrococcaceae; g__Kocuria | 0.046771 | 0.0496794 |
| k__Bacteria; p__Firmicutes; c__Bacilli; o__Bacillales; f__Bacillaceae; g__Virgibacillus; s__ | 0.046927 | 0.0495883 |
| k__Bacteria; p__Verrucomicrobia; c__Verrucomicrobiae; o__Verrucomicrobiales; f__Verrucomicrobiaceae; g__; s__ | 0.047148 | 0.0495661 |
| k__Bacteria; p__Firmicutes; c__Clostridia; o__Clostridiales; f__Ruminococcaceae; g__Oscillospira; s__ | 0.04743 | 0.0496081 |
| k__Bacteria; p__Proteobacteria; c__Gammaproteobacteria; o__Thiotrichales; f__Thiotrichaceae; g__Leucothrix; s__ | 0.047671 | 0.0496067 |
| k__Bacteria; p__Proteobacteria; c__Gammaproteobacteria; o__Pseudomonadales; f__Moraxellaceae; g__Acinetobacter | 0.047911 | 0.0496053 |
| k__Bacteria; p__Proteobacteria; c__Gammaproteobacteria; o__Pseudomonadales; f__Moraxellaceae; g__Acinetobacter; s__guillouiae | 0.048024 | 0.0494722 |
| k__Bacteria; p__Firmicutes; c__Clostridia; o__Clostridiales | 0.048551 | 0.0497651 |
| k__Bacteria; p__Proteobacteria; c__Gammaproteobacteria; o__Pseudomonadales; f__Moraxellaceae; g__Acinetobacter | 0.048665 | 0.0496331 |
| k__Bacteria; p__Firmicutes; c__Bacilli; o__Bacillales; f__Bacillaceae | 0.048701 | 0.0494241 |
| k__Bacteria; p__Firmicutes; c__Bacilli; o__Lactobacillales; f__Streptococcaceae; g__Streptococcus; s__agalactiae | 0.049024 | 0.0495067 |
| k__Bacteria; p__Proteobacteria; c__Betaproteobacteria; o__Burkholderiales; f__Comamonadaceae | 0.049095 | 0.0493361 |
| k__Bacteria; p__Tenericutes; c__Mollicutes; o__Anaeroplasmatales; f__Anaeroplasmataceae; g__Anaeroplasma; s__ | 0.04935 | 0.0493501 |
