# Supplementary material for: Differences in airway microbiome and metabolome of single lung transplant recipients
Source: Respir Res. 2020 May 6;21:104. doi: 10.1186/s12931-020-01367-3 (PMC7201609; doi:10.1186/s12931-020-01367-3)
Supplement: Supplementary file 3 — Additional file 3. [file 12931_2020_1367_MOESM3_ESM.docx]

| ConsensusLineage | P value | FDR P value |
| --- | --- | --- |
| k__Bacteria; p__Firmicutes; c__Clostridia; o__Clostridiales; f__Lachnospiraceae; g__Dorea; s__ | 4.96E-06 | 0.001011183 |
| k__Bacteria; p__Firmicutes; c__Clostridia; o__Clostridiales; f__Ruminococcaceae; g__Oscillospira; s__ | 0.000113 | 0.011541708 |
| k__Bacteria; p__Firmicutes; c__Clostridia; o__Clostridiales | 0.000124 | 0.008450836 |
| k__Bacteria; p__Proteobacteria; c__Gammaproteobacteria; o__Pseudomonadales; f__Moraxellaceae; g__Acinetobacter | 0.000167 | 0.008535615 |
| k__Bacteria; p__Cyanobacteria; c__Chloroplast; o__Rhodophyta; f__; g__; s__ | 0.000355 | 0.0144636 |
| k__Bacteria; p__Proteobacteria; c__Gammaproteobacteria; o__Pseudomonadales; f__Moraxellaceae; g__Acinetobacter; s__ | 0.000432 | 0.014699832 |
| k__Bacteria; p__Bacteroidetes; c__Bacteroidia; o__Bacteroidales; f__Bacteroidaceae; g__Bacteroides | 0.000445 | 0.012963792 |
| k__Bacteria; p__Proteobacteria; c__Gammaproteobacteria; o__Pseudomonadales; f__Moraxellaceae; g__Acinetobacter | 0.000458 | 0.011689583 |
| k__Bacteria; p__Proteobacteria; c__Gammaproteobacteria; o__Thiotrichales; f__Thiotrichaceae; g__Cocleimonas; s__ | 0.000506 | 0.011473504 |
| k__Bacteria; p__Firmicutes; c__Clostridia; o__Clostridiales | 0.000616 | 0.012575009 |
| k__Bacteria; p__Proteobacteria; c__Gammaproteobacteria; o__Pseudomonadales; f__Moraxellaceae; g__Acinetobacter | 0.000624 | 0.011565372 |
| k__Bacteria; p__Bacteroidetes; c__Bacteroidia; o__Bacteroidales; f__Bacteroidaceae; g__Bacteroides | 0.000631 | 0.010719367 |
| k__Bacteria; p__Proteobacteria; c__Gammaproteobacteria; o__Pseudomonadales; f__Pseudomonadaceae; g__Pseudomonas; s__ | 0.000674 | 0.010579456 |
| k__Bacteria; p__Proteobacteria; c__Gammaproteobacteria; o__Pseudomonadales; f__Pseudomonadaceae | 0.000712 | 0.010374639 |
| k__Bacteria; p__Firmicutes; c__Bacilli; o__Lactobacillales; f__Lactobacillaceae; g__Lactobacillus; s__ | 0.000715 | 0.009718057 |
| k__Bacteria; p__Proteobacteria; c__Gammaproteobacteria; o__; f__; g__; s__ | 0.000716 | 0.009129434 |
| k__Bacteria; p__Proteobacteria; c__Gammaproteobacteria; o__Pseudomonadales; f__Moraxellaceae; g__Acinetobacter | 0.000733 | 0.008790912 |
| k__Bacteria; p__Proteobacteria; c__Gammaproteobacteria; o__Pseudomonadales; f__Moraxellaceae; g__Acinetobacter | 0.000767 | 0.008697925 |
| k__Bacteria; p__Firmicutes; c__Clostridia; o__Clostridiales; f__Ruminococcaceae; g__Anaerotruncus; s__ | 0.000817 | 0.008771721 |
| k__Bacteria; p__Proteobacteria; c__Gammaproteobacteria; o__Pseudomonadales; f__Moraxellaceae; g__Acinetobacter | 0.000838 | 0.008550293 |
| k__Bacteria; p__Bacteroidetes; c__Flavobacteriia; o__Flavobacteriales; f__Flavobacteriaceae | 0.000886 | 0.008609635 |
| k__Bacteria; p__Firmicutes; c__Bacilli; o__Lactobacillales; f__Lactobacillaceae; g__Lactobacillus; s__iners | 0.001029 | 0.009540755 |
| k__Bacteria; p__Actinobacteria; c__Actinobacteria; o__Bifidobacteriales; f__Bifidobacteriaceae; g__Bifidobacterium | 0.001189 | 0.010541939 |
| k__Bacteria; p__Firmicutes; c__Bacilli; o__Bacillales; f__Bacillaceae | 0.001423 | 0.012095279 |
| k__Bacteria; p__Proteobacteria; c__Gammaproteobacteria; o__Alteromonadales; f__[Chromatiaceae]; g__; s__ | 0.001452 | 0.011845676 |
| k__Bacteria; p__Bacteroidetes; c__Bacteroidia; o__Bacteroidales; f__Bacteroidaceae; g__Bacteroides; s__caccae | 0.001493 | 0.011711499 |
| k__Bacteria; p__Fusobacteria; c__Fusobacteriia; o__Fusobacteriales; f__Fusobacteriaceae; g__Propionigenium; s__ | 0.001494 | 0.011286481 |
| k__Bacteria; p__Proteobacteria; c__Gammaproteobacteria; o__Pseudomonadales; f__Moraxellaceae; g__Acinetobacter | 0.001497 | 0.010910277 |
| k__Bacteria; p__Firmicutes; c__Clostridia; o__Clostridiales; f__Lachnospiraceae; g__Coprococcus; s__ | 0.00151 | 0.010620184 |
| k__Bacteria; p__Proteobacteria; c__Alphaproteobacteria; o__Sphingomonadales; f__Sphingomonadaceae; g__Sphingomonas | 0.001589 | 0.010802636 |
| k__Bacteria; p__Proteobacteria; c__Gammaproteobacteria; o__Pseudomonadales; f__Moraxellaceae; g__Acinetobacter | 0.001734 | 0.011414024 |
| k__Bacteria; p__Proteobacteria; c__Gammaproteobacteria; o__Pseudomonadales; f__Moraxellaceae; g__Acinetobacter | 0.001821 | 0.011610864 |
| k__Bacteria; p__Bacteroidetes; c__Bacteroidia; o__Bacteroidales; f__S24-7; g__; s__ | 0.001889 | 0.01167484 |
| k__Bacteria; p__Bacteroidetes; c__Bacteroidia; o__Bacteroidales; f__Porphyromonadaceae; g__Parabacteroides; s__ | 0.0019 | 0.011399832 |
| k__Bacteria; p__Verrucomicrobia; c__Verrucomicrobiae; o__Verrucomicrobiales; f__Verrucomicrobiaceae; g__; s__ | 0.001948 | 0.01135594 |
| k__Bacteria; p__Proteobacteria; c__Gammaproteobacteria; o__Pseudomonadales; f__Moraxellaceae; g__Acinetobacter; s__ | 0.001962 | 0.011116946 |
| k__Bacteria; p__Proteobacteria; c__Gammaproteobacteria; o__Pseudomonadales; f__Moraxellaceae; g__Acinetobacter | 0.002173 | 0.011979757 |
| k__Bacteria; p__Proteobacteria; c__Gammaproteobacteria; o__Pseudomonadales; f__Moraxellaceae; g__Acinetobacter | 0.002182 | 0.011712504 |
| k__Bacteria; p__Bacteroidetes; c__Bacteroidia; o__Bacteroidales; f__Porphyromonadaceae; g__Porphyromonas; s__ | 0.0023 | 0.012031031 |
| k__Bacteria; p__Bacteroidetes; c__Flavobacteriia; o__Flavobacteriales; f__; g__; s__ | 0.002451 | 0.012499498 |
| k__Bacteria; p__Proteobacteria; c__Gammaproteobacteria; o__Pseudomonadales; f__Moraxellaceae; g__Acinetobacter | 0.002586 | 0.012866932 |
| k__Bacteria; p__Proteobacteria; c__Gammaproteobacteria; o__Pseudomonadales; f__Moraxellaceae; g__Acinetobacter | 0.002752 | 0.013369116 |
| k__Bacteria; p__Bacteroidetes; c__[Saprospirae]; o__[Saprospirales]; f__Saprospiraceae; g__; s__ | 0.002776 | 0.01317136 |
| k__Bacteria; p__Proteobacteria; c__Gammaproteobacteria; o__Pseudomonadales; f__Pseudomonadaceae; g__Pseudomonas; s__fragi | 0.002835 | 0.013146024 |
| k__Bacteria; p__Firmicutes; c__Bacilli; o__Lactobacillales; f__Lactobacillaceae; g__Lactobacillus; s__ | 0.00284 | 0.01287575 |
| k__Bacteria; p__Cyanobacteria; c__Chloroplast; o__Streptophyta; f__; g__; s__ | 0.002868 | 0.012719391 |
| k__Bacteria; p__Bacteroidetes; c__Bacteroidia; o__Bacteroidales; f__[Odoribacteraceae]; g__Odoribacter; s__ | 0.003072 | 0.01333268 |
| k__Bacteria; p__Actinobacteria; c__Actinobacteria; o__Bifidobacteriales; f__Bifidobacteriaceae; g__Bifidobacterium | 0.003205 | 0.013621174 |
| k__Bacteria; p__Proteobacteria; c__Gammaproteobacteria; o__Pseudomonadales; f__Moraxellaceae; g__Acinetobacter; s__ | 0.003345 | 0.013927496 |
| k__Bacteria; p__Proteobacteria; c__Gammaproteobacteria; o__Pseudomonadales; f__Moraxellaceae; g__; s__ | 0.003401 | 0.013878014 |
| k__Bacteria; p__Bacteroidetes; c__Bacteroidia; o__Bacteroidales; f__Bacteroidaceae; g__Bacteroides | 0.003509 | 0.014034864 |
| k__Bacteria; p__Proteobacteria; c__Gammaproteobacteria; o__Pseudomonadales; f__Moraxellaceae; g__Acinetobacter | 0.003719 | 0.014590209 |
| k__Bacteria; p__Proteobacteria; c__Betaproteobacteria; o__Neisseriales; f__Neisseriaceae; g__Neisseria | 0.003792 | 0.014595665 |
| k__Bacteria; p__Proteobacteria; c__Gammaproteobacteria; o__Pseudomonadales; f__Moraxellaceae | 0.003856 | 0.01456744 |
| k__Bacteria; p__Proteobacteria; c__Gammaproteobacteria; o__Pseudomonadales; f__Moraxellaceae | 0.003925 | 0.014559395 |
| k__Bacteria; p__Firmicutes; c__Clostridia; o__Clostridiales; f__Lachnospiraceae | 0.004036 | 0.014702721 |
| k__Bacteria; p__Firmicutes; c__Clostridia; o__Clostridiales; f__Lachnospiraceae | 0.004217 | 0.015092203 |
| k__Bacteria; p__Proteobacteria; c__Betaproteobacteria; o__Burkholderiales; f__Alcaligenaceae; g__Sutterella; s__ | 0.004326 | 0.01521699 |
| k__Bacteria; p__Firmicutes; c__Erysipelotrichi; o__Erysipelotrichales; f__Erysipelotrichaceae; g__[Eubacterium]; s__dolichum | 0.004383 | 0.015153082 |
| k__Bacteria; p__Bacteroidetes; c__Flavobacteriia; o__Flavobacteriales; f__Flavobacteriaceae; g__Maribacter; s__ | 0.004425 | 0.015043419 |
| k__Bacteria; p__Proteobacteria; c__Gammaproteobacteria; o__Pseudomonadales; f__Moraxellaceae; g__Acinetobacter; s__ | 0.004431 | 0.014818614 |
| k__Bacteria; p__Proteobacteria; c__Gammaproteobacteria; o__Pseudomonadales; f__Moraxellaceae; g__Acinetobacter | 0.004438 | 0.014603633 |
| k__Bacteria; p__Firmicutes; c__Clostridia; o__Clostridiales; f__[Tissierellaceae]; g__Peptoniphilus; s__ | 0.004785 | 0.015494104 |
| k__Bacteria; p__Firmicutes; c__Clostridia; o__Clostridiales; f__Clostridiaceae; g__SMB53; s__ | 0.005089 | 0.016222319 |
| k__Bacteria; p__Proteobacteria; c__Gammaproteobacteria; o__Enterobacteriales; f__Enterobacteriaceae | 0.005526 | 0.017341874 |
| k__Bacteria; p__Proteobacteria; c__Gammaproteobacteria; o__Pseudomonadales; f__Moraxellaceae; g__Acinetobacter | 0.005639 | 0.017428524 |
| k__Bacteria; p__Proteobacteria; c__Gammaproteobacteria; o__Pseudomonadales; f__Moraxellaceae; g__Acinetobacter | 0.005829 | 0.017746831 |
| k__Bacteria; p__Proteobacteria; c__Gammaproteobacteria; o__Pseudomonadales; f__Moraxellaceae; g__Acinetobacter | 0.005841 | 0.017522331 |
| k__Bacteria; p__Proteobacteria; c__Gammaproteobacteria; o__Alteromonadales; f__Colwelliaceae; g__; s__ | 0.005896 | 0.017432752 |
| k__Bacteria; p__Actinobacteria; c__Acidimicrobiia; o__Acidimicrobiales; f__JdFBGBact; g__; s__ | 0.006121 | 0.017837416 |
| k__Bacteria; p__Verrucomicrobia; c__Verrucomicrobiae; o__Verrucomicrobiales; f__Verrucomicrobiaceae; g__Akkermansia; s__muciniphila | 0.006285 | 0.018059657 |
| k__Bacteria; p__Proteobacteria; c__Gammaproteobacteria; o__Alteromonadales; f__Colwelliaceae; g__Thalassomonas | 0.006358 | 0.018015339 |
| k__Bacteria; p__Firmicutes; c__Clostridia; o__Clostridiales | 0.006595 | 0.018429651 |
| k__Bacteria; p__Bacteroidetes; c__Bacteroidia; o__Bacteroidales; f__S24-7; g__; s__ | 0.006605 | 0.018207802 |
| k__Bacteria; p__Firmicutes; c__Bacilli; o__Lactobacillales; f__Streptococcaceae; g__Streptococcus; s__luteciae | 0.00669 | 0.01819733 |
| k__Bacteria; p__Firmicutes; c__Clostridia; o__Clostridiales; f__Veillonellaceae; g__Megasphaera; s__ | 0.0068 | 0.018252932 |
| k__Bacteria; p__Proteobacteria; c__Gammaproteobacteria; o__Pseudomonadales; f__Moraxellaceae; g__Acinetobacter | 0.006998 | 0.018540733 |
| k__Bacteria; p__Bacteroidetes; c__Bacteroidia; o__Bacteroidales; f__S24-7; g__; s__ | 0.007031 | 0.018387663 |
| k__Bacteria; p__Proteobacteria; c__Betaproteobacteria; o__Burkholderiales; f__Alcaligenaceae; g__Sutterella; s__ | 0.007187 | 0.018559058 |
| k__Bacteria; p__Actinobacteria; c__Actinobacteria; o__Actinomycetales; f__Corynebacteriaceae; g__Corynebacterium; s__ | 0.007294 | 0.018599833 |
| k__Bacteria; p__Bacteroidetes; c__Bacteroidia; o__Bacteroidales; f__Rikenellaceae; g__AF12; s__ | 0.007416 | 0.018676792 |
| k__Bacteria; p__Bacteroidetes; c__Bacteroidia; o__Bacteroidales; f__Prevotellaceae; g__Prevotella; s__ | 0.007553 | 0.018791216 |
| k__Bacteria; p__Firmicutes; c__Clostridia; o__Clostridiales; f__; g__; s__ | 0.007752 | 0.019053602 |
| k__Bacteria; p__Proteobacteria; c__Gammaproteobacteria; o__Pseudomonadales; f__Moraxellaceae; g__Acinetobacter | 0.007815 | 0.018980468 |
| k__Bacteria; p__Bacteroidetes; c__Bacteroidia; o__Bacteroidales; f__Porphyromonadaceae; g__Porphyromonas; s__ | 0.007939 | 0.019052604 |
| k__Bacteria; p__Firmicutes; c__Clostridia; o__Clostridiales; f__Veillonellaceae; g__Megasphaera; s__ | 0.008098 | 0.019209781 |
| k__Bacteria; p__Bacteroidetes; c__Bacteroidia; o__Bacteroidales; f__S24-7; g__; s__ | 0.008204 | 0.019237824 |
| k__Bacteria; p__Proteobacteria; c__Gammaproteobacteria; o__Pseudomonadales; f__Pseudomonadaceae | 0.008541 | 0.019800381 |
| k__Bacteria; p__Proteobacteria; c__Gammaproteobacteria; o__Pseudomonadales; f__Moraxellaceae; g__Acinetobacter | 0.008639 | 0.019801673 |
| k__Bacteria; p__Proteobacteria; c__Alphaproteobacteria; o__BD7-3; f__; g__; s__ | 0.008672 | 0.019656567 |
| k__Bacteria; p__Proteobacteria; c__Gammaproteobacteria; o__Pseudomonadales; f__Moraxellaceae; g__Acinetobacter | 0.008897 | 0.019945555 |
| k__Bacteria; p__Proteobacteria; c__Gammaproteobacteria; o__Pseudomonadales; f__Moraxellaceae; g__Acinetobacter | 0.008935 | 0.019812875 |
| k__Bacteria; p__Bacteroidetes; c__Bacteroidia; o__Bacteroidales; f__Bacteroidaceae; g__Bacteroides; s__acidifaciens | 0.009537 | 0.020919908 |
| k__Bacteria; p__Proteobacteria; c__Gammaproteobacteria; o__Alteromonadales; f__Colwelliaceae; g__; s__ | 0.009815 | 0.021300068 |
| k__Bacteria; p__Bacteroidetes; c__Bacteroidia; o__Bacteroidales; f__Bacteroidaceae; g__Bacteroides | 0.009861 | 0.021174732 |
| k__Bacteria; p__Proteobacteria; c__Deltaproteobacteria; o__Desulfovibrionales; f__Desulfovibrionaceae; g__Desulfovibrio; s__C21_c20 | 0.010129 | 0.021523545 |
| k__Bacteria; p__Fusobacteria; c__Fusobacteriia; o__Fusobacteriales; f__Leptotrichiaceae; g__Sneathia; s__ | 0.010473 | 0.022025167 |
| k__Bacteria; p__Firmicutes; c__Bacilli; o__Lactobacillales; f__Lactobacillaceae; g__Lactobacillus; s__ | 0.010584 | 0.022032445 |
| k__Bacteria; p__Bacteroidetes; c__Bacteroidia; o__Bacteroidales; f__[Paraprevotellaceae]; g__[Prevotella]; s__ | 0.011064 | 0.022797548 |
| k__Bacteria; p__Proteobacteria; c__Gammaproteobacteria; o__Thiotrichales; f__Thiotrichaceae; g__Leucothrix; s__ | 0.011394 | 0.023244029 |
| k__Bacteria; p__Proteobacteria; c__Gammaproteobacteria; o__Pseudomonadales; f__Moraxellaceae; g__Acinetobacter; s__guillouiae | 0.0114 | 0.023024894 |
| k__Bacteria; p__Tenericutes; c__Mollicutes; o__Mycoplasmatales; f__Mycoplasmataceae; g__Mycoplasma; s__ | 0.011409 | 0.022817632 |
| k__Bacteria; p__Actinobacteria; c__Actinobacteria; o__Actinomycetales; f__Propionibacteriaceae; g__Propionibacterium; s__acnes | 0.011499 | 0.022774305 |
| k__Bacteria | 0.011848 | 0.023240561 |
| k__Bacteria; p__Bacteroidetes; c__Bacteroidia; o__Bacteroidales; f__Bacteroidaceae; g__Bacteroides; s__ | 0.012412 | 0.024113837 |
| k__Bacteria; p__Proteobacteria; c__Gammaproteobacteria; o__Pseudomonadales; f__Moraxellaceae; g__Acinetobacter; s__johnsonii | 0.012835 | 0.024700511 |
| k__Bacteria; p__Bacteroidetes; c__Bacteroidia; o__Bacteroidales | 0.01293 | 0.02465127 |
| k__Bacteria; p__Bacteroidetes; c__Flavobacteriia; o__Flavobacteriales; f__Flavobacteriaceae; g__Flavobacterium; s__succinicans | 0.013156 | 0.024849399 |
| k__Bacteria; p__Proteobacteria; c__Gammaproteobacteria; o__Enterobacteriales; f__Enterobacteriaceae; g__Escherichia; s__coli | 0.013225 | 0.024751266 |
| k__Bacteria; p__Bacteroidetes; c__Bacteroidia; o__Bacteroidales; f__Prevotellaceae; g__Prevotella; s__ | 0.013323 | 0.02470782 |
| k__Bacteria; p__Bacteroidetes; c__Bacteroidia; o__Bacteroidales; f__S24-7; g__; s__ | 0.01406 | 0.025840039 |
| k__Bacteria; p__Firmicutes; c__Bacilli; o__Lactobacillales; f__Lactobacillaceae; g__Lactobacillus; s__iners | 0.014186 | 0.025839345 |
| k__Bacteria; p__Firmicutes; c__Clostridia; o__Clostridiales; f__; g__; s__ | 0.014305 | 0.025824486 |
| k__Bacteria; p__Proteobacteria; c__Deltaproteobacteria; o__Desulfovibrionales; f__Desulfovibrionaceae; g__; s__ | 0.014365 | 0.025705278 |
| k__Bacteria; p__Proteobacteria; c__Betaproteobacteria; o__Neisseriales; f__Neisseriaceae; g__Neisseria; s__ | 0.014378 | 0.025504935 |
| k__Bacteria; p__Firmicutes; c__Clostridia; o__Clostridiales; f__Lachnospiraceae | 0.014437 | 0.025389968 |
| k__Bacteria; p__Proteobacteria; c__Gammaproteobacteria; o__Pseudomonadales; f__Moraxellaceae; g__Acinetobacter | 0.014677 | 0.025590168 |
| k__Bacteria; p__Proteobacteria; c__Gammaproteobacteria; o__Xanthomonadales; f__Xanthomonadaceae; g__Stenotrophomonas; s__ | 0.015568 | 0.026914394 |
| k__Bacteria; p__Proteobacteria; c__Gammaproteobacteria; o__Pseudomonadales; f__Moraxellaceae; g__Acinetobacter | 0.015598 | 0.026739489 |
| k__Bacteria; p__Bacteroidetes; c__Bacteroidia; o__Bacteroidales; f__S24-7; g__; s__ | 0.015994 | 0.027189538 |
| k__Bacteria; p__Firmicutes; c__Bacilli; o__Lactobacillales; f__Lactobacillaceae; g__Lactobacillus | 0.016219 | 0.027344389 |
| k__Bacteria; p__Bacteroidetes; c__Bacteroidia; o__Bacteroidales; f__S24-7; g__; s__ | 0.016464 | 0.027530282 |
| k__Bacteria; p__Firmicutes; c__Clostridia; o__Clostridiales; f__[Tissierellaceae]; g__WAL_1855D; s__ | 0.016503 | 0.027370086 |
| k__Bacteria; p__Fusobacteria; c__Fusobacteriia; o__Fusobacteriales; f__Leptotrichiaceae; g__Leptotrichia; s__ | 0.016593 | 0.027298943 |
| k__Bacteria; p__Bacteroidetes; c__Bacteroidia; o__Bacteroidales; f__[Paraprevotellaceae]; g__[Prevotella]; s__ | 0.016809 | 0.027432343 |
| k__Bacteria; p__Bacteroidetes; c__Bacteroidia; o__Bacteroidales; f__S24-7; g__; s__ | 0.016844 | 0.027271476 |
| k__Bacteria; p__Bacteroidetes; c__[Saprospirae]; o__[Saprospirales]; f__Saprospiraceae; g__; s__ | 0.017551 | 0.028192263 |
| k__Bacteria; p__Firmicutes; c__Clostridia; o__Clostridiales; f__Veillonellaceae; g__Veillonella; s__ | 0.017928 | 0.028573244 |
| k__Bacteria; p__Bacteroidetes; c__Bacteroidia; o__Bacteroidales; f__S24-7; g__; s__ | 0.018047 | 0.028539709 |
| k__Bacteria; p__Proteobacteria; c__Betaproteobacteria; o__Burkholderiales; f__Alcaligenaceae; g__Sutterella; s__ | 0.018364 | 0.028816688 |
| k__Bacteria; p__Firmicutes; c__Clostridia; o__Clostridiales; f__[Tissierellaceae]; g__Anaerococcus; s__ | 0.018515 | 0.028833052 |
| k__Bacteria; p__Proteobacteria; c__Gammaproteobacteria; o__Pseudomonadales; f__Moraxellaceae; g__Acinetobacter; s__ | 0.019337 | 0.029883686 |
| k__Bacteria; p__Bacteroidetes; c__Flavobacteriia; o__Flavobacteriales; f__Flavobacteriaceae | 0.019505 | 0.029917102 |
| k__Bacteria; p__Firmicutes; c__Clostridia; o__Clostridiales | 0.019744 | 0.030058611 |
| k__Bacteria; p__Firmicutes; c__Clostridia; o__Clostridiales; f__[Tissierellaceae] | 0.019752 | 0.029848113 |
| k__Bacteria; p__Bacteroidetes; c__Bacteroidia; o__Bacteroidales; f__S24-7; g__; s__ | 0.019873 | 0.02980933 |
| k__Bacteria; p__Bacteroidetes; c__Bacteroidia; o__Bacteroidales; f__S24-7; g__; s__ | 0.020128 | 0.029971975 |
| k__Bacteria; p__Bacteroidetes; c__Bacteroidia; o__Bacteroidales; f__Prevotellaceae; g__Prevotella; s__ | 0.020133 | 0.029762494 |
| k__Bacteria; p__Actinobacteria; c__Actinobacteria; o__Actinomycetales; f__Micrococcaceae; g__Rothia; s__dentocariosa | 0.020717 | 0.030404669 |
| k__Bacteria; p__Proteobacteria; c__Gammaproteobacteria; o__Pasteurellales; f__Pasteurellaceae; g__Haemophilus; s__parainfluenzae | 0.020791 | 0.03029577 |
| k__Bacteria; p__Proteobacteria; c__Gammaproteobacteria; o__Pseudomonadales; f__Moraxellaceae; g__Acinetobacter | 0.021048 | 0.030452343 |
| k__Bacteria; p__Bacteroidetes; c__Bacteroidia; o__Bacteroidales; f__Bacteroidaceae; g__Bacteroides; s__ovatus | 0.021724 | 0.031209351 |
| k__Bacteria; p__Verrucomicrobia; c__Verrucomicrobiae; o__Verrucomicrobiales; f__Verrucomicrobiaceae; g__Akkermansia; s__muciniphila | 0.02243 | 0.031997624 |
| k__Bacteria; p__Proteobacteria; c__Gammaproteobacteria; o__Alteromonadales; f__Psychromonadaceae; g__Psychromonas; s__ | 0.022567 | 0.031970574 |
| k__Bacteria; p__Proteobacteria; c__Gammaproteobacteria; o__Pseudomonadales; f__Moraxellaceae; g__Acinetobacter; s__ | 0.02259 | 0.031781382 |
| k__Bacteria; p__Bacteroidetes; c__Bacteroidia; o__Bacteroidales; f__S24-7; g__; s__ | 0.022866 | 0.031949174 |
| k__Bacteria; p__Actinobacteria; c__Acidimicrobiia; o__Acidimicrobiales; f__JdFBGBact; g__; s__ | 0.023083 | 0.032033257 |
| k__Bacteria; p__Bacteroidetes; c__Bacteroidia; o__Bacteroidales; f__Bacteroidaceae; g__Bacteroides; s__ | 0.023636 | 0.032579397 |
| k__Bacteria; p__Proteobacteria; c__Gammaproteobacteria; o__Pseudomonadales; f__Pseudomonadaceae; g__Pseudomonas | 0.023697 | 0.032444683 |
| k__Bacteria; p__Proteobacteria; c__Alphaproteobacteria; o__Rhodobacterales; f__Rhodobacteraceae; g__Octadecabacter | 0.02416 | 0.032858011 |
| k__Bacteria; p__Proteobacteria; c__Gammaproteobacteria | 0.024557 | 0.033176993 |
| k__Bacteria; p__Proteobacteria; c__Gammaproteobacteria; o__Pasteurellales; f__Pasteurellaceae; g__Actinobacillus | 0.025261 | 0.033902918 |
| k__Bacteria; p__Bacteroidetes; c__Bacteroidia; o__Bacteroidales; f__Bacteroidaceae; g__Bacteroides | 0.025424 | 0.033899236 |
| k__Bacteria; p__Firmicutes; c__Clostridia; o__Clostridiales | 0.026059 | 0.034519447 |
| k__Bacteria; p__Proteobacteria; c__Betaproteobacteria; o__Burkholderiales; f__Comamonadaceae | 0.026216 | 0.03450381 |
| k__Bacteria; p__Firmicutes; c__Bacilli; o__Lactobacillales; f__Enterococcaceae; g__Enterococcus | 0.026289 | 0.034378515 |
| k__Bacteria; p__Proteobacteria; c__Gammaproteobacteria | 0.02667 | 0.034653475 |
| k__Bacteria; p__Proteobacteria; c__Gammaproteobacteria; o__Pseudomonadales; f__Moraxellaceae; g__Acinetobacter | 0.027058 | 0.0349353 |
| k__Bacteria; p__Proteobacteria; c__Gammaproteobacteria; o__Alteromonadales; f__Shewanellaceae; g__Shewanella; s__ | 0.027188 | 0.034882537 |
| k__Bacteria; p__Firmicutes; c__Clostridia; o__Clostridiales | 0.027314 | 0.034824795 |
| k__Bacteria; p__Firmicutes; c__Bacilli; o__Turicibacterales; f__Turicibacteraceae; g__Turicibacter; s__ | 0.027919 | 0.03537573 |
| k__Bacteria; p__Proteobacteria; c__Deltaproteobacteria; o__Desulfovibrionales; f__Desulfovibrionaceae; g__Bilophila; s__ | 0.028634 | 0.036057631 |
| k__Bacteria; p__Bacteroidetes | 0.02871 | 0.035931795 |
| k__Bacteria; p__Tenericutes; c__Mollicutes; o__Mycoplasmatales; f__Mycoplasmataceae; g__Ureaplasma; s__ | 0.029014 | 0.036090327 |
| k__Bacteria; p__Firmicutes; c__Clostridia; o__Clostridiales; f__[Tissierellaceae]; g__Anaerococcus; s__ | 0.029021 | 0.035880847 |
| k__Bacteria; p__Actinobacteria; c__Actinobacteria; o__Actinomycetales; f__Corynebacteriaceae; g__Corynebacterium; s__kroppenstedtii | 0.029634 | 0.03641721 |
| k__Bacteria; p__Firmicutes; c__Bacilli; o__Bacillales; f__Alicyclobacillaceae; g__Alicyclobacillus; s__ | 0.03004 | 0.036695875 |
| k__Bacteria; p__Bacteroidetes; c__Bacteroidia; o__Bacteroidales; f__Bacteroidaceae; g__Bacteroides; s__ | 0.030486 | 0.037018571 |
| k__Bacteria; p__Proteobacteria; c__Betaproteobacteria; o__Burkholderiales; f__Burkholderiaceae; g__Burkholderia; s__ | 0.030575 | 0.036907272 |
| k__Bacteria; p__Proteobacteria; c__Gammaproteobacteria; o__Vibrionales; f__Vibrionaceae | 0.03105 | 0.03726042 |
| k__Bacteria; p__Bacteroidetes; c__Bacteroidia; o__Bacteroidales; f__S24-7; g__; s__ | 0.03118 | 0.037197309 |
| k__Bacteria; p__Proteobacteria; c__Betaproteobacteria; o__Rhodocyclales; f__Rhodocyclaceae; g__Hydrogenophilus; s__ | 0.031375 | 0.037212065 |
| k__Bacteria; p__Firmicutes; c__Bacilli; o__Bacillales; f__Bacillaceae | 0.031792 | 0.037489087 |
| k__Bacteria; p__Proteobacteria; c__Gammaproteobacteria; o__Pseudomonadales; f__Moraxellaceae; g__Acinetobacter | 0.032623 | 0.03824781 |
| k__Bacteria; p__Firmicutes; c__Clostridia; o__Clostridiales; f__Veillonellaceae; g__Veillonella; s__ | 0.033025 | 0.038498287 |
| k__Bacteria; p__Firmicutes; c__Erysipelotrichi; o__Erysipelotrichales; f__Erysipelotrichaceae; g__Allobaculum; s__ | 0.033249 | 0.038539034 |
| k__Bacteria; p__Bacteroidetes; c__Bacteroidia; o__Bacteroidales; f__Prevotellaceae; g__Prevotella | 0.033607 | 0.038733013 |
| k__Bacteria; p__Bacteroidetes; c__Bacteroidia; o__Bacteroidales; f__[Odoribacteraceae]; g__Odoribacter; s__ | 0.034405 | 0.039430518 |
| k__Bacteria | 0.036002 | 0.041029758 |
| k__Bacteria; p__Firmicutes; c__Bacilli; o__Lactobacillales; f__Streptococcaceae; g__Streptococcus; s__ | 0.0372 | 0.042159842 |
| k__Bacteria; p__Verrucomicrobia; c__Verrucomicrobiae; o__Verrucomicrobiales; f__Verrucomicrobiaceae; g__Akkermansia; s__muciniphila | 0.037484 | 0.042247129 |
| k__Bacteria; p__Proteobacteria; c__Gammaproteobacteria; o__Alteromonadales; f__Moritellaceae; g__Moritella; s__ | 0.038242 | 0.042864901 |
| k__Bacteria; p__Firmicutes; c__Clostridia; o__Clostridiales; f__Dehalobacteriaceae; g__Dehalobacterium; s__ | 0.038534 | 0.04295594 |
| k__Bacteria; p__Firmicutes; c__Clostridia; o__Clostridiales; f__Lachnospiraceae | 0.038621 | 0.042819468 |
| k__Bacteria; p__Fusobacteria; c__Fusobacteriia; o__Fusobacteriales; f__Leptotrichiaceae; g__Sneathia; s__ | 0.039298 | 0.043334442 |
| k__Bacteria; p__Firmicutes; c__Clostridia; o__Clostridiales; f__Lachnospiraceae | 0.039395 | 0.043207958 |
| k__Bacteria; p__Bacteroidetes; c__Bacteroidia; o__Bacteroidales; f__S24-7; g__; s__ | 0.039777 | 0.043392549 |
| k__Bacteria; p__Proteobacteria; c__Betaproteobacteria; o__Rhodocyclales; f__Rhodocyclaceae | 0.040262 | 0.04368891 |
| k__Bacteria; p__Firmicutes; c__Clostridia; o__Clostridiales; f__Lachnospiraceae; g__[Ruminococcus]; s__gnavus | 0.041196 | 0.044465788 |
| k__Bacteria; p__Actinobacteria; c__Actinobacteria; o__Bifidobacteriales; f__Bifidobacteriaceae; g__Bifidobacterium | 0.042149 | 0.045255175 |
| k__Bacteria; p__Proteobacteria; c__Gammaproteobacteria; o__Alteromonadales; f__Psychromonadaceae; g__Psychromonas; s__ | 0.042309 | 0.045188776 |
| k__Bacteria; p__Tenericutes; c__Mollicutes; o__Mycoplasmatales; f__Mycoplasmataceae; g__Mycoplasma; s__genitalium | 0.042328 | 0.044973181 |
| k__Bacteria; p__Actinobacteria; c__Actinobacteria; o__Actinomycetales; f__Micrococcaceae; g__Micrococcus; s__ | 0.042751 | 0.045187187 |
| k__Bacteria; p__Proteobacteria; c__Gammaproteobacteria; o__Pseudomonadales; f__Moraxellaceae; g__Acinetobacter; s__guillouiae | 0.043572 | 0.045817481 |
| k__Bacteria; p__Actinobacteria; c__Actinobacteria; o__Actinomycetales; f__Actinomycetaceae | 0.044013 | 0.046044021 |
| k__Bacteria; p__Bacteroidetes; c__Bacteroidia; o__Bacteroidales; f__Prevotellaceae; g__Prevotella; s__ | 0.044249 | 0.04605479 |
| k__Bacteria; p__Firmicutes; c__Clostridia; o__Clostridiales | 0.044539 | 0.046121229 |
| k__Bacteria; p__Firmicutes; c__Clostridia; o__Clostridiales; f__[Tissierellaceae]; g__Peptoniphilus; s__ | 0.045692 | 0.047077103 |
| k__Bacteria; p__Proteobacteria; c__Gammaproteobacteria | 0.047014 | 0.04819573 |
| k__Bacteria; p__Firmicutes; c__Clostridia; o__Clostridiales | 0.047353 | 0.048300273 |
| k__Bacteria; p__Proteobacteria; c__Gammaproteobacteria; o__Pseudomonadales; f__Moraxellaceae; g__Acinetobacter; s__guillouiae | 0.047439 | 0.04814673 |
| k__Bacteria; p__Actinobacteria; c__Actinobacteria; o__Actinomycetales; f__Micrococcaceae; g__Sinomonas; s__ | 0.049035 | 0.049520849 |
| k__Bacteria; p__Bacteroidetes; c__Bacteroidia; o__Bacteroidales; f__S24-7; g__; s__ | 0.049181 | 0.049423065 |
| k__Bacteria; p__Firmicutes; c__Clostridia; o__Clostridiales; f__Ruminococcaceae; g__Oscillospira; s__ | 0.049446 | 0.049446126 |
