# Supplementary material for: Differences in airway microbiome and metabolome of single lung transplant recipients
Source: Respir Res. 2020 May 6;21:104. doi: 10.1186/s12931-020-01367-3 (PMC7201609; doi:10.1186/s12931-020-01367-3)
Supplement: Supplementary file 7 — Additional file 7. [file 12931_2020_1367_MOESM7_ESM.docx]

**SUPPLEMENTARY FIGURES**

**
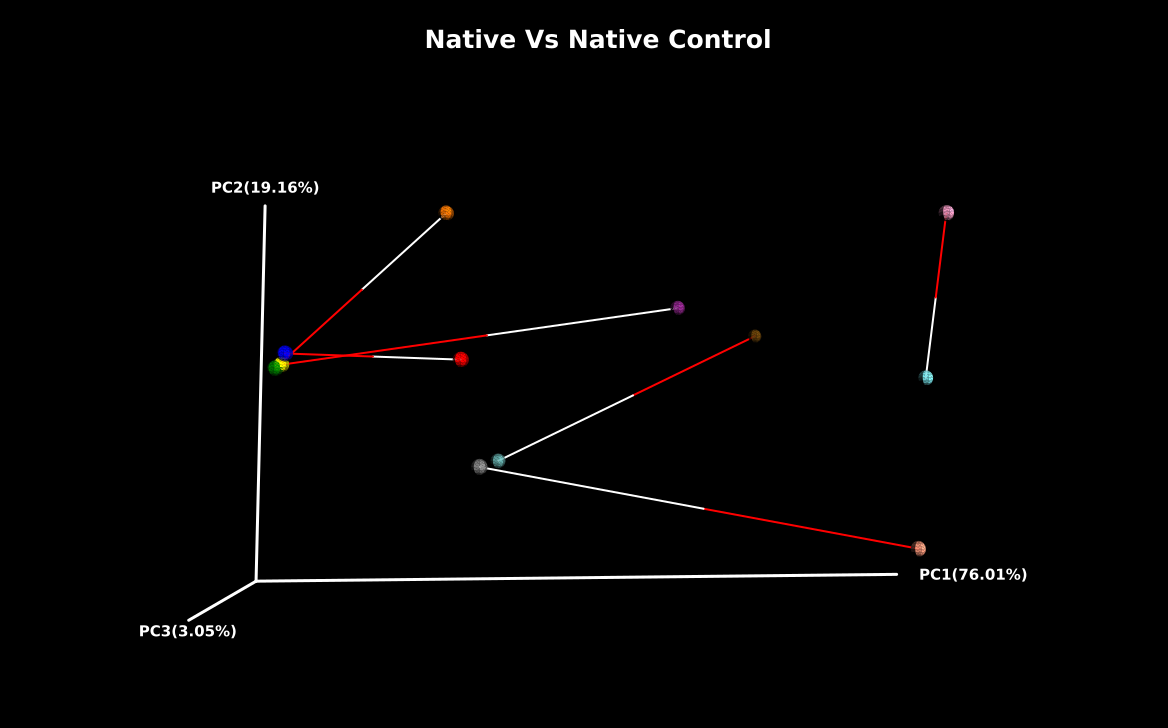
**

Native versus Native Control

**Figure S3**

**Figure S1A. Principal component analysis showing Weighted Unifrac distance between bronchoscopic control and airway microbiome lung transplant recipients.** Relationship between Native (N) and bronchoscopic control sample (NC) bacterial communities within individual subjects. Weighted UniFrac distances were calculated between all pairs of samples within N or NC, and then each sample type was plotted separately in 3D space by principal coordinate analysis. The two plots (N and NC) were then transformed by Procrustes analysis to achieve maximum alignment. Each point corresponds to a bacterial community, with NC communities are joined at the white end of each bar; the *red* end connects to the N sample data from the same individual. If N and NC plots are similar, then the relative distance between connected points (residuals) will be small. The overall similarity is summarized by the M^2^ value, and statistical goodness of fit is measured by a Monte Carlo label permutation approach (10,000 iterations). The M^2^ value ranges from 0-1, with 0 suggesting complete overlap i.e. similarity and 1 suggesting maximum variation

**
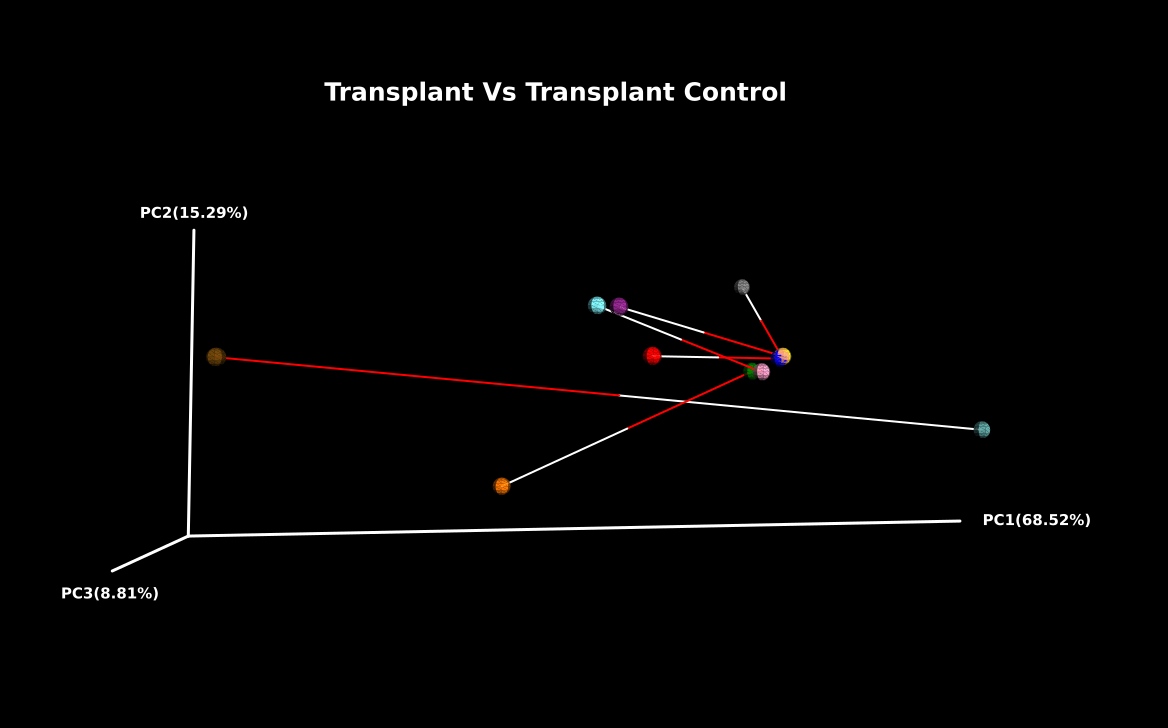
**

Transplant versus Transplant Control

**Figure S1B. Principal component analysis showing Weighted Unifrac distance between bronchoscopic control and airway microbiome lung transplant recipients.** Relationship between Allograft (A) and bronchoscopic control samples (AC) bacterial communities within individual subjects. Weighted UniFrac distances were calculated between all pairs of samples within A or AC, and then each sample type was plotted separately in 3D space by principal coordinate analysis. The two plots (A and AC) were then transformed by Procrustes analysis to achieve maximum alignment. Each point corresponds to a bacterial community, with AC communities joined at the *white* end of each bar; the red end connects to the A sample data from the same individual. If A and AC plots are similar, then the relative distance between connected points (residuals) will be small. The overall similarity is summarized by the M^2^ value, and statistical goodness of fit is measured by a Monte Carlo label permutation approach (10,000 iterations). The M^2^ value ranges from 0-1, with 0 suggesting complete overlap i.e. similarity and 1 suggesting maximum variation.

**Figure S2A. LTB4 levels in BAL from native and allograft lungs in single lung transplant recipients. P value was calculated using non-parametric Wilcoxon test. P**

**Figure S2B. IL-8 levels in BAL from native and allograft lungs in single lung transplant recipients. P value was calculated using non-parametric Wilcoxon test. P=0.07.**

**
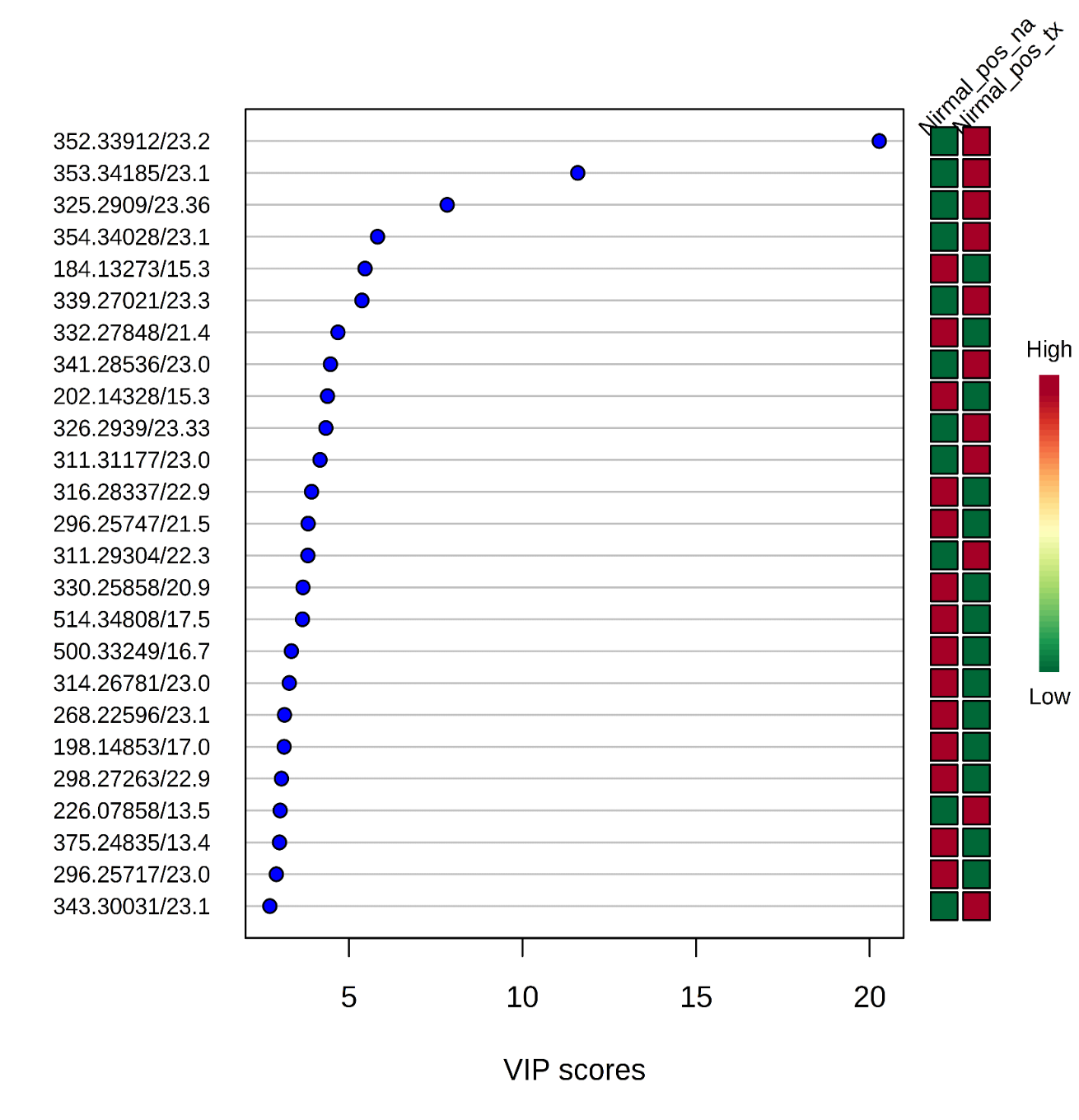
**

Feature (*m/z, RT)*

VIP SCORES

A

N

Positive Mode

**Figure S3A. Top variable importance in projection (VIP) score metabolite features in positive and negative mode from allograft and native lung samples.** Top VIP score in the positive mode with Y-axis showing the m/z (mass/charge)/ RT (retention time) feature and X-axis showing VIP scores**.** Legend shows gradient showing abundance of metabolites with green being low and red showing high metabolite concentration in the sample. N represents native and A, the allograft sample. **Those highlighted in red bar are m/z features identified as sphingosine like molecules.**

**
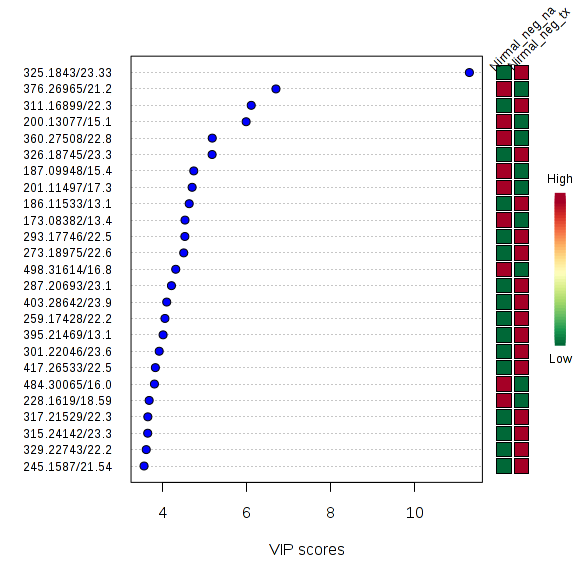
**

Negative Mode

N

A

VIP SCORES

Feature (*m/z, RT)*

**Figure S3B. Top variable importance in projection (VIP) score metabolite features in positive and negative mode from allograft and native lung samples**

1. Top VIP score in the negative mode with Y-axis showing the m/z feature)/ RT (retention time) and X-axis showing VIP scores**.** Legend shows gradient showing abundance of metabolites with green being low and red showing high metabolite concentration in the sample. N represents native and A, the allograft sample.
