# Supplementary material for: Differences in airway microbiome and metabolome of single lung transplant recipients
Source: Respir Res. 2020 May 6;21:104. doi: 10.1186/s12931-020-01367-3 (PMC7201609; doi:10.1186/s12931-020-01367-3)
Supplement: Supplementary file 9 — Additional file 9. [file 12931_2020_1367_MOESM9_ESM.docx]

**Online Data Supplement**

**Section I: Methods**

**A. Isolation of Microbial DNA and Creation of 16S V4 amplicon Library.**

Microbial genomic DNA was isolated using the Fecal DNA isolation kit (Zymo Research, Irvine, CA) following the manufacturer’s instructions. Once the sample DNA was prepared, PCR was used with unique bar coded primers to amplify the V4 region of the 16S rRNA gene to create an “amplicon library” from individual samples (Kozich et. al, Kumar et. al.)

**5’PRIMER:** 5’AATGATACGGCGACCACCGAGATCTACACTATGGTAATTGTGTGCCAGCMGCCGCGGTAA 3’

**3’ PRIMER:**

5’CAAGAGAAGACGGCATACGAGATNNNNNNAGTCAGTCAGCCGGACTACHVGGGTWTCTAAT3’

The primers were synthesized at 50nmol scale with desalting purification (Eurofins-mwg-operon, Huntsville, AL). The primers were diluted with 10 mM Tris pH 8.0 to 100 µM, then diluted 10X in water to 10µM for use in PCR reactions. The individual PCR reactions were set up as follows (Kumar et. al.):

10 µL of 5X Reaction Buffer

1.5µLof dNTPs

2µL of the 5 ‘Primer diluted as described above

2 µL of the 3’ Primer diluted as described above

1.5 µL of the “LongAmp” enzyme kit (New England Biolabs LongAmp Taq PCR kit,

30 µL of the Template DNA prepared using the “Fecal DNA Isolation kit with the concentration of DNA at 2-5 ng/ul.

3µL of H_2_O

The total reaction volume is 50 µL

Cycling conditions for the PCR were as follows:

Initial denature 94^o^ C 1 minute

32 cycles of:

94^o^C 30 seconds

50^o^C 1 minute

65^o^C 1 minute

Final extension: 65^o^C 3 minutes

Final hold: 4^o^ C

Following PCR, the entire PCR reaction was electrophoresed on a 1.0% agarose/Tris-borate-EDTA gel. The PCR product (approximately 380 base pair predicted product size) was visualized by UV illumination. The band was excised and purified from the agarose using QIAquick Gel Extraction Kit according to manufacturer’s instructions. (Qiagen, Valencia, CA).

**DNA sequencing.**

The PCR products were sequenced using NextGen sequencing Illumina MiSeq platform (Kozich et al., Kumar et. al.). The MiSeq is a single flowcell, single lane instrument that can generate approximately 9Gb of sequence data from a paired end 250bp run (Caparoso et. al.). The paired 250 base pair end kits from Illumina were used for the V4 region in the microbiome analysis. The samples were first quantitated using Pico Green, adjusted to a concentration of 4nM and then used for sequencing on the Illumina MiSeq (Kumar et. al.).

Fastq conversion of the raw data files was performed following de-multiplexing. Quality control of the fastq files was performed then subject to quality assessment and filtering using the FASTX toolkit (FASTX). The remainder of the steps was performed with the Quantitative Insight into Microbial Ecology (QIIME) suite, version 1.7. (Lozupone et. al., Kumar et al., Navas-Molina et. al.)

**Bioinformatics.**

The sequence data covered the 16S rRNA V4 region with a PCR product length of ~255 bases and 250 base paired-end reads. Since the overlap between fragments was approximately 245 bases, the information from both ends of the paired reads was merged to generate a single high quality read using the module “fastq_mergepairs” of USEARCH (Edgar RC). Read pairs with an overlap of less than 50 bases or with too many mismatches (> 20) in the overlapping region were discarded. Chimeric sequences were also filtered using the “identify_chimeric_seqs.py” module of USEARCH (Edgar RC). Overall read quality was assessed before and after filtering using FASTQC (FASTQC). The QIIME data analysis package was used for subsequent 16S rRNA data analysis (Caparoso et al.). Sequences were grouped into operational taxonomic units (OTUs) using the clustering program UCLUST at a similarity threshold of 0.97% (Edgar RC). The Ribosomal Database Program (RDP) classifier was used to make taxonomic assignments (to the species level) for all OTUs at confidence threshold of 60% (0.6) (Wang et al.). The RDP classifier was trained using the Greengenes (v13_8) 16S rRNA database (McDonald,et al.). The resulting OTU table included all OTUs, their taxonomic identification, and abundance information. OTUs whose average abundance was less than 0.0005% were filtered out. OTUs were then grouped together to summarize taxon abundance at different hierarchical levels of classification (e.g. phylum, class, order, family, genus, and species). These taxonomy tables were also used to generate bar charts of taxon abundance. Multiple sequence alignment of OTUs was performed with PyNAST (Caporaso, et al. 2010a). Alpha diversity (within sample diversity) was calculated using a variety of diversity metrics including Shannon’s, Chao1, and Simpson, as implemented in QIIME (Caporaso, et al. 2010b). Beta diversity (between sample diversity) among different samples was measured using Unifrac analysis (Lozupone et al 2006). Principal coordinates analysis (PCoA) was performed by QIIME to visualize the dissimilarity matrix (beta-diversity) between all the samples, such that samples that are more similar are closer in space than samples that are more divergent. 3D PCoA plots were generated using EMPEROR (Vázquez-Baeza Y et al.). A heatmap with the top 50 most highly abundant taxa across all samples was generated using the “heatmap.2” function in R package (available at [http://CRAN.R-project.org/package=gplots](http://cran.r-project.org/package=gplots)).

**B. PGP Measurement By Electrospray Ionization–Liquid Chromatography–Tandem Mass Spectrometry (ESI-LC/MS/MS)**

PGP peptides in human BAL were quantified by isotope dilution, liquid chromatography-electrospray ionization-multiple reaction monitoring (LC-MRM) using a Shimadzu LC system and a SCIEX API-4000 triple quadrupole mass spectrometer. Analysis was performed a 2.0 × 150–mm Jupiter 4μ Proteo column (Phenomenex). The column was equilibrated with solvent A (0.1% ([v/v]) formic acid in water). PGP and acetyl-PGP were eluted by solvent B (0.1% formic acid in acetonitrile) using gradient elution: 0 to 0.5 min 5% B/95% A, then increased linearly over 0.5 to 2.5 min to 100% B/0% A. Prior to each run, samples were kept at 4°C. The column temperature was at 30°C. Positive electrospray mass transitions were *m/z* (mass to-charge ratio) 312 to 140, *m/z* 312 to 112, and *m/z* 312 to 70  for Ac-PGP and *m/z* 270 to 70, *m/z* 270 to 116, and *m/z* 270 to 173 for PGP. Peak areas were measured, and PGP peptide concentrations were calculated using a relative standard curve method as previously described [9].

**C. Metabolomics**

**Extraction of Lung Lavage**

Methanol (800 ml, cooled to -20^o^C) was added to each lung lavage sample (200 μL). After mixing, the samples were allowed to stand at –20^o^C for 30 min. They were centrifuged at 12,000 x g for 10 min at 4^o^C to remove precipitated protein. Supernatants were transferred to a clean glass tube and dried down under N_2_. The dried samples were re-suspended in 100 μL of 0.1% formic acid in ddH_2_O and centrifuged again at 12,000 x g for 10 minutes at 4^o^C. The supernatants were transferred to a fresh 1.5 mL micro centrifuge tube and stored at -80^o^C until assayed.

**Global Metabolite LC-MS/MS Analysis**

An aliquot (5 μL) of each sample was loaded onto a Nano cHiPLC 200 μm ID x 6 mm ChromXP C_18_-CL 3 μm, 120 Å reverse-phase trap cartridge (Eksigent, Dublin,CA) at 2 μL/min using an Eksigent autosampler. After washing the cartridge for 5 min with 0.1% formic acid in double-distilled water, the bound metabolites were flushed onto an analytical Nano cHiPLC column (200 μm ID x 15 cm ChromXP C_18_-CL with a 20 min, linear gradient of 5-95% acetonitrile in 0.1% formic acid at 1000 nl/min using an Eksigent 415 NanoLC system. The column was washed with 95% acetonitrile-0.1% formic acid for 5 min and then re-equilibrated with 5% acetonitrile-0.1% formic acid for 5 min. The SCIEX 5600 Triple-Tof mass spectrometer (SCIEX, Concord, Ontario, Canada) was used to analyze the metabolite profile. The IonSpray™ voltages for positive and negative modes were +/-2300 V, respectively, and the declustering potential was +/- 80 V. Ionspray™ and curtain gases were set at 10 psi and 25 psi, respectively. The interface heater temperature was 120^o^C.

Eluted compounds were subjected to a time-of-flight (TOF) survey scan from *m/z* 50-1000 to determine the top twenty most intense ions for MSMS analysis. During the duty cycle (1.25 s), product ion TOF scans to obtain the tandem mass spectra of the selected parent ions over the range from *m/z* 50-1000 were collected over 50 msec intervals using a collision energy spread of 15 eV with a set collision point of 35 eV. Spectra were centroided and de-isotoped by Analyst software, version 1.6 TF (SCIEX).

**Data Analysis and Metabolite Identification**

LC-MS data were processed using XCMSonline (<https://xcmsonline.scripps.edu/>) to detect and align peak occurring across all samples. The *m/z* values and retention times of each peak and their areas were downloaded as an Excel .xlsx file. Individual Excel .csv files for each sample were created containing the *m/z* value, peak area and retention time of each metabolite ion. These files were placed in folders corresponding to the two groups and the folders converted to single .zip file. The zip file was uploaded to Metaboanalyst 4.0 ( ) (<http://www.metaboanalyst.ca)>. To take into account variable dilution of the BAL, the data were normalized to the total ion current of each sample. Further, the data were mean-centered and subjected to Pareto scaling. Statistical procedures were used to generate Volcano plots (univariate analysis) and sparse PLS-DA plots and Variable in Projection (VIP) scores for the ions contributing most to the separation of the two groups (multivariate analysis). After verifying the peaks in the VIP list, their identity was assessed using METLIN (<https://metlin.scripps.edu/)> using both MS and, where available, MSMS data. The normalized data were used to create a .txt file containing the *m/z*, retention time, p-value and t-score for each ion. This file was submitted for pathway analysis using Mummichog, version 1.0.9 (XX).

**D. Cytokine/VEGF assay**

TNF-α, IFN-γ and VEGF in BAL fluid were assayed by a luminex magnetic bead based multiplexed (Biorad Bio-Plex 200 system, Luminex Bio, USA) assay using commercially available kits (Miliplex MAP Human cytokine /chemokine, Cat# HCYTOMAGA-60K) according to the manufacturer's recommended protocol.

**E. Bacterial quantification via 16S qPCR**

To generate quantification curves, purified DNA from Pseudomonas aeruginosa was quantified using a Qubit Fluorometric estimation (Thermo Fisher Scientific). This DNA was subsequently diluted serially by copy number (calculated by molecular weight) and amplified using the 16S rRNA qRT-PCR assay.

A standard curve of ct value vs copy number was plotted using the serially diluted samples using RT PCR and finally the bacterial load in the BAL fluid were extrapolated from the curve.

16S Primers used were 5’- GCAGGCCTAACACATGCAAGTC-3’ (63F) and 5’- CTGCTGCCTCCCGTAGGAGT-3’ (355R). The cycling protocol was 1 cycle at 95°C for 5 minutes, 40 cycles at 95°C for 15 seconds and 60°C for 1 minute, 1 cycle at 4°C for 5 minutes, and 1 cycle at 90°C for 5 minutes all at a ramp rate of 2°C/second. The BioRad C1000 Touch Thermal Cycler was used for PCR cycling. Three replicates were used per sample. Negative control specimens were used and were run alongside lung specimens. The bacterial burden in bronchoalveolar lavage specimens were reported as total bacterial 16S gene copies per mL of lavage fluid.

**Demographics Table for Non-Transplant Controls**

| Age | Gender | Current smoker | Known Lung disease | FEV1PP |
| --- | --- | --- | --- | --- |
| 68 | M | N | NA | Not available |
| 46 | F | N | NA | Not available |
| 43 | M | N | NA | 102 |
| 55 | F | N | NA | 119 |

**References**

Caporaso, J.G., Bittinger, K., Bushman, F.D., DeSantis, T.Z., Andersen, G.L., and Knight, R. 2010a. PyNAST: a flexible tool for aligning sequences to a template alignment. *Bioinformatics (Oxford, England)* 26:266-267.

Caporaso, J.G., Kuczynski, J., Stombaugh, J., Bittinger, K., Bushman, F.D., Costello, E.K., Fierer, N., Pena, A.G., Goodrich, J.K., Gordon, J.I., Huttley, G.A., Kelley, S.T., Knights, D., Koenig, J.E., Ley, R.E., Lozupone, C.A., McDonald, D., Muegge, B.D., Pirrung, M., Reeder, J., Sevinsky, J.R., Turnbaugh, P.J., Walters, W.A., Widmann, J., Yatsunenko, T., Zaneveld, J., and Knight, R. 2010b. QIIME allows analysis of high-throughput community sequencing data. *Nature methods* 7:335-336.

Caporaso, J.G., Lauber, C.L., Walters, W.A., Berg-Lyons, D., Huntley, J., Fierer, N., Owens, S.M., Betley, J., Fraser, L., Bauer, M., Gormley, N., Gilbert, J.A., Smith, G., and Knight, R. 2012. Ultra-high-throughput microbial community analysis on the Illumina HiSeq and MiSeq platforms. *The ISME journal* 6:1621-1624.

Edgar, R.C. 2010. Search and clustering orders of magnitude faster than BLAST. Bioinformatics (Oxford, England) 26:2460-2461.

FASTX <http://hannonlab.cshl.edu/fastx_toolkit/>

FASTQC (<http://www.bioinformatics.babraham.ac.uk/projects/fastqc/>)

Kozich, J.J., Westcott, S.L., Baxter, N.T., Highlander, S.K., and Schloss, P.D. 2013. Development of a dual-index sequencing strategy and curation pipeline for analyzing amplicon sequence data on the MiSeq Illumina sequencing platform. *Applied and environmental microbiology* 79:5112-5120.

Kumar, R., Eipers, P., Little, R.B., Crowley, M., Crossman, D.K., Lefkowitz, E. J. and Morrow, C.D. 2014. Getting Started with Microbiome Analysis: Sample Acquisition to Bioinformatics. *Current Protocols in Human Genetics.* 18.8.1-18.8.28, July 2014 Published online July 2014 in Wiley Online Library (wileyonlinelibrary.com). DOI: 10.1002/0471142905.hg1808s82

Lozupone, C., Hamady, M., and Knight, R. 2006. UniFrac--an online tool for comparing microbial community diversity in a phylogenetic context. *BMC bioinformatics* 7:371.

Lozupone, C.A., Hamady, M., Kelley, S.T., and Knight, R. 2007. Quantitative and qualitative beta diversity measures lead to different insights into factors that structure microbial communities. *Applied and environmental microbiology* 73:1576-1585.

McDonald, D., Price, M.N., Goodrich, J., Nawrocki, E.P., DeSantis, T.Z., Probst, A., Andersen, G.L., Knight, R., and Hugenholtz, P. 2012. An improved Greengenes taxonomy with explicit ranks for ecological and evolutionary analyses of bacteria and archaea. *The ISME journal* 6:610-618.

Navas-Molina, J.A., Peralta-Sanchez, J.M., Gonzalez, A., McMurdie, P.J., Vazquez-Baeza, Y., Xu, Z., Ursell, L.K., Lauber, C., Zhou, H., Song, S.J., Huntley, J., Ackermann, G.L., Berg-Lyons, D., Holmes, S., Caporaso, J.G., and Knight, R. 2013. Advancing Our Understanding of the Human Microbiome Using QIIME. *Methods in enzymology* 531:371-444.

Vázquez-Baeza Y, Pirrung M, Gonzalez A, Knight R. 2013 [EMPeror: a tool for visualizing high-throughput microbial community data.](http://www.ncbi.nlm.nih.gov/pubmed/24280061) GigaScience. 2:16.

Wang, Q., Garrity, G.M., Tiedje, J.M., and Cole, J.R. 2007. Naive Bayesian classifier for rapid assignment of rRNA sequences into the new bacterial taxonomy. *Applied and environmental microbiology* 73:5261-5267.
