# Supplementary material for: Differences in airway microbiome and metabolome of single lung transplant recipients
Source: Respir Res. 2020 May 6;21:104. doi: 10.1186/s12931-020-01367-3 (PMC7201609; doi:10.1186/s12931-020-01367-3)
Supplement: Supplementary file 9 [file 12931_2020_1367_MOESM1_ESM.docx]

Table ST1. Differences between the airway microbiome of Native and Allograft Lung of Single Lung Transplant Recipients. (FDR cutoff 0.1)

#OTU ID	ConsensusLineage	P Value (paired T test)	FDR P value
denovo10618	k__Bacteria; p__Proteobacteria; c__Alphaproteobacteria; o__Caulobacterales; f__Caulobacteraceae; g__; s__	0.003009813	0.111363081
denovo23187	k__Bacteria; p__Proteobacteria; c__Gammaproteobacteria; o__Pseudomonadales; f__Moraxellaceae; g__; s__	0.008599292	0.159086902
denovo21583	k__Bacteria; p__Proteobacteria; c__Gammaproteobacteria; o__Pseudomonadales; f__Moraxellaceae; g__Acinetobacter	0.008779438	0.108279735
denovo57755	k__Bacteria; p__Proteobacteria; c__Gammaproteobacteria; o__Pseudomonadales; f__Moraxellaceae; g__Acinetobacter	0.009521814	0.08807678
denovo65068	k__Bacteria; p__Proteobacteria; c__Gammaproteobacteria; o__Pseudomonadales; f__Moraxellaceae; g__Acinetobacter	0.009573694	0.070845336
denovo70729	k__Bacteria; p__Proteobacteria; c__Gammaproteobacteria; o__Pseudomonadales; f__Moraxellaceae; g__Acinetobacter	0.01036271	0.063903378
denovo65795	k__Bacteria; p__Proteobacteria; c__Gammaproteobacteria; o__Pseudomonadales; f__Moraxellaceae; g__Acinetobacter	0.010872694	0.057469954
denovo44315	k__Bacteria; p__Proteobacteria; c__Gammaproteobacteria; o__Pseudomonadales; f__Pseudomonadaceae	0.014781863	0.068366116
denovo16239	k__Bacteria; p__Proteobacteria; c__Gammaproteobacteria; o__Pseudomonadales; f__Pseudomonadaceae; g__Pseudomonas; s__	0.019245829	0.079121741
denovo60588	k__Bacteria; p__Proteobacteria; c__Gammaproteobacteria; o__Pseudomonadales; f__Pseudomonadaceae; g__Pseudomonas; s__fragi	0.020976566	0.077613294
denovo38771	k__Bacteria; p__Proteobacteria; c__Gammaproteobacteria; o__Pseudomonadales; f__Moraxellaceae; g__Acinetobacter	0.021961165	0.073869373
denovo58337	k__Bacteria; p__Proteobacteria; c__Gammaproteobacteria; o__Pseudomonadales; f__Moraxellaceae; g__Acinetobacter; s__	0.025700889	0.079244408
denovo46242	k__Bacteria; p__Proteobacteria; c__Betaproteobacteria; o__Burkholderiales; f__Oxalobacteraceae	0.027164994	0.077315752
denovo6052	k__Bacteria; p__Proteobacteria; c__Gammaproteobacteria; o__Enterobacteriales; f__Enterobacteriaceae	0.027951385	0.073871518
denovo69049	k__Bacteria; p__Proteobacteria; c__Gammaproteobacteria; o__Pseudomonadales; f__Pseudomonadaceae	0.028460502	0.070202572
denovo20583	k__Bacteria; p__Actinobacteria; c__Actinobacteria; o__Actinomycetales; f__Micrococcaceae; g__Rothia; s__mucilaginosa	0.029375724	0.067931362
denovo32429	k__Bacteria; p__Proteobacteria; c__Gammaproteobacteria; o__Pseudomonadales; f__Moraxellaceae; g__Acinetobacter	0.030464018	0.066304039
denovo52933	k__Bacteria; p__Proteobacteria; c__Gammaproteobacteria; o__Pseudomonadales; f__Moraxellaceae; g__Acinetobacter	0.031381641	0.064506707
denovo23028	k__Bacteria; p__Proteobacteria; c__Gammaproteobacteria; o__Pseudomonadales; f__Moraxellaceae; g__Acinetobacter	0.032328343	0.062955194
denovo66701	k__Bacteria; p__Proteobacteria; c__Gammaproteobacteria; o__Pseudomonadales; f__Moraxellaceae; g__; s__	0.032912023	0.060887243
denovo79567	k__Bacteria; p__Proteobacteria; c__Gammaproteobacteria; o__Pseudomonadales; f__Moraxellaceae; g__Acinetobacter	0.033013518	0.058166675
denovo76888	k__Bacteria; p__Proteobacteria; c__Gammaproteobacteria; o__Pseudomonadales; f__Moraxellaceae; g__Acinetobacter	0.033552849	0.056429792
denovo25924	k__Bacteria; p__Bacteroidetes; c__Bacteroidia; o__Bacteroidales; f__S24-7; g__; s__	0.034245844	0.05509114
denovo4723	k__Bacteria; p__Firmicutes; c__Bacilli; o__Lactobacillales; f__Streptococcaceae; g__Streptococcus; s__anginosus	0.035808134	0.055204207
denovo49899	k__Bacteria; p__Proteobacteria; c__Gammaproteobacteria; o__Pseudomonadales; f__Moraxellaceae; g__Acinetobacter	0.036546525	0.054088857
denovo34056	k__Bacteria; p__Firmicutes; c__Clostridia; o__Clostridiales; f__Veillonellaceae; g__Veillonella; s__dispar	0.039432824	0.056115942
denovo13740	k__Bacteria; p__Proteobacteria; c__Gammaproteobacteria; o__Pseudomonadales; f__Moraxellaceae; g__Acinetobacter; s__	0.039994556	0.054807355
denovo19127	k__Bacteria; p__Proteobacteria; c__Gammaproteobacteria; o__Pseudomonadales; f__Moraxellaceae; g__Acinetobacter	0.040026203	0.052891768
denovo85089	k__Bacteria; p__Proteobacteria; c__Gammaproteobacteria; o__Pseudomonadales; f__Moraxellaceae; g__Acinetobacter	0.040968902	0.052270668
denovo81026	k__Bacteria; p__Proteobacteria; c__Betaproteobacteria; o__Burkholderiales; f__Oxalobacteraceae	0.040985685	0.050549012
denovo80114	k__Bacteria; p__Proteobacteria; c__Gammaproteobacteria; o__Pseudomonadales; f__Moraxellaceae; g__Acinetobacter	0.041346437	0.049348973
denovo57963	k__Bacteria; p__Firmicutes; c__Bacilli; o__Lactobacillales; f__Streptococcaceae; g__Streptococcus; s__	0.043413982	0.050197417
denovo7176	k__Bacteria; p__Proteobacteria; c__Gammaproteobacteria; o__Pseudomonadales; f__Moraxellaceae; g__Acinetobacter	0.044744971	0.050168604
denovo75514	k__Bacteria; p__Firmicutes; c__Clostridia; o__Clostridiales; f__Veillonellaceae; g__Megasphaera; s__	0.045342034	0.049342802
denovo43846	k__Bacteria; p__Proteobacteria; c__Gammaproteobacteria; o__Enterobacteriales; f__Enterobacteriaceae	0.04675815	0.049430044
denovo73945	k__Bacteria; p__Proteobacteria; c__Gammaproteobacteria; o__Pseudomonadales; f__Moraxellaceae; g__Acinetobacter; s__	0.048575229	0.049924541
denovo54339	k__Bacteria; p__Proteobacteria; c__Gammaproteobacteria; o__Pseudomonadales; f__Moraxellaceae; g__Acinetobacter	0.049830047	0.049830047
